# Identification of novel candidate genes for the inverted teat defect in sows using a genome-wide marker panel

**DOI:** 10.1007/s13353-016-0382-1

**Published:** 2017-01-04

**Authors:** Helena Chalkias, Elisabeth Jonas, Lisa S. Andersson, Magdalena Jacobson, Dirk Jan de Koning, Nils Lundeheim, Gabriella Lindgren

**Affiliations:** 1 Department of Animal Breeding and Genetics, Swedish University of Agricultural Sciences, Box 7023, SE-750 07 Uppsala, Sweden; 2 Department of Clinical Sciences, Swedish University of Agricultural Sciences, Box 7054, SE-750 07 Uppsala, Sweden; 3 Capilet Genetics AB, SE-725 93 Västerås, Sweden

**Keywords:** Genome-wide association study, SNP, Genetic markers, Yorkshire breed, Teat number, Functional teats

## Abstract

The number of functional teats is an important selection criterion in pig breeding. Inherited defects of the udder, such as the inverted teat, do have a considerable negative impact on the nursing ability of the sow. To investigate the genetic background of this defect and the number of functional teats in Swedish maternal lines, samples from 230 Yorkshire pigs were selected for genotyping using the PorcineSNP60K BeadChip (Illumina Inc.), each pig with at least one inverted teat was matched with one non-affected pig (fullsib or pairs with matching herd and gender). A genome-wide association study on these 230 pigs was performed using the two-step approach implemented in GenABEL using 46,652 single nucleotide polymorphisms across all autosomes and the X chromosome. A number of significant regions were identified for the inverted teat defect on chromosomes 2, 10, and 18. Many of the regions associated with the number of functional teats were located in the same or close regions, except two associated markers on the X chromosome and one on chromosome 3. We identified some of the regions on chromosomes previously reported in one linkage and one gene expression study. We conclude, despite being able to suggest new candidate genes, that further studies are needed to better understand the biologic background of the teat development. Despite the in-depth comparison of identified regions for the inverted teat defect done here, more studies are required to allow a clear identification of genetic regions relevant for this defect across many pig populations.

**Electronic supplementary material:**

The online version of this article (doi:10.1007/s13353-016-0382-1) contains supplementary material, which is available to authorized users.

## Introduction

The successful breeding for increased litter sizes has made the number of functional teats a critical trait for selection for increased litter size in pigs. If the number of teats is lower than the number of piglets, the piglet mortality might be increased. Cross-fostering might to some degree even out the lack of a functional teat for each piglet. Nevertheless, the selection pressure on breeding sows with a healthy udder and many functional teats is high. A common type of inherited non-functional teat is the inverted teat. It has been shown that, while some inverted teats protrude just before or during the lactation, those that remain inverted cannot be suckled by the piglet (Chalkias et al. 2014). However, as each piglet should have access to its own teat for the supplement of colostrum and survival during the nursing period, and as piglets usually develop a rank order at the udder early on, a non-functional teat can lead to significant disturbance of the nursing (Chalkias et al. 2014). One problem is that inverted teats are difficult to distinguish from normal teats in the young gilt at the stage of selection, and phenotypic selection therefore remains difficult (Chalkias et al. 2013). The frequency of inverted teats differs between pig breeds and populations. It has been shown that about 12% of the performance-tested pigs of the Yorkshire population in Sweden have non-functional teats (Chalkias et al. 2013). Selection for functional teats is therefore relevant, but will take ‘selection space’ from other traits in the breeding goal. Also, as traditional selection has not been able to eradicate inverted teats from the pig populations, further studies on the genetic background should assist in reducing this inherited defect.

Presently, only a few studies have focused on the identification of genetic regions associated with this trait, using linkage and expression analysis (Chomwisarutkun et al. 2012a, b; Jonas et al. 2008). A number of candidate genes had been tested, including parathyroid hormone-like hormone gene (*PTHLH*) and its receptor (*PTHR1*), which have been suggested as possible regulators for inverted teats (Tetzlaff et al. 2009). However, none of these genetic variations were able to explain the larger proportion of the phenotypic variation of this trait. Furthermore, these studies have been limited to pig lines in Germany.

The aim of this study was therefore to identify novel chromosomal regions and markers associated with the number of inverted teats, the number of functional teats, and the total number of teats in Swedish Yorkshire pigs using a dense marker panel. This study was also performed to investigate if the previously identified genomic region using German pig lines could be identified also in the Swedish pig cohort.

## Materials and methods

### Animals and phenotypes

The pigs included in our study were part of the Nordic Genetics breeding scheme for Yorkshire sows. The pigs were selected based on the routine teat assessment at the performance testing done at 100 kg live weight. We collected blood samples from pigs with at least one inverted (case) teat, and if possible samples from one full sibling with no inverted teat (control). A total of 230 animals from seven different nucleus herds were included in the study. The samples from 100 full sib pairs and 15 unrelated pairs were collected between 2008 and 2012. Phenotypic records used in the analyses were total number of inverted teats, number of functional teats, and total number of teats, recorded at approximately 5 months of age.

Technicians from the breeding company Nordic Genetics (www.nordicgenetics.com) scored, as part of the performance testing, young pigs (at approximately 100 kg and 5 months of age) for the total number of teats (left + right side), number of functional teats, and number of non-functional teats (including inverted teats). Pedigree, phenotypic, and management (technician identity) information were stored in the Nordic Genetics database. Additional information from the database including pedigree, herd, and date of birth, as well as the technician scoring the animals, were used for the further analysis. More details on the population and scoring as well as results of heritability estimations can be found in a previous publication (Chalkias et al. 2013).

### Genotyping

Blood samples were collected in EDTA tubes from the jugular vein of each pig in connection with regular performance testing in the nucleus herds when the pigs weighed approximate 100 kg. DNA was extracted from blood samples of 230 selected pigs using the QIAGEN QIAsymphony^SP^ Midi kit (www.qiagen.com). The DNA concentration was measured using a Nano Drop 8000 spectrophotometer (Thermo Scientific). The samples were genotyped with the Illumina PorcineSNP60K BeadChip (Illumina, San Diego, CA, USA) containing 61,565 SNPs. Quality control was performed; markers and samples were removed due to low call rate (<95%) and minor allele frequency (<5%). A total of 23% of data were excluded prior to the association analysis. and 46,652 markers remained for the association analysis.

### Association analysis

Possible subpopulations were estimated using identity by state (IBS) distance clustering. To identify population stratification in the sample population, quantile–quantile plots (QQ plots) were used. The genome-wide association analysis was performed in R (version 2.15.1; http://CRAN.R-project.org) using the GRAMMAR–gamma method (Amin et al. 2007; Aulchenko et al. 2007a; Chen and Abecasis 2007) implemented in the GenABEL software (version 1.7-4) (Aulchenko et al. 2007b). This analysis is suitable for the analysis of data from sib-pairs without parentsm while avoiding false-positive results (Boehnke and Langefeld 1998). The GRAMMAR–gamma approach derives the residuals with a linear mixed model, and treats the residuals in a second step as phenotypes to test the marker effect (Svishcheva et al. 2012). The traits used for the association analysis were total number of teats, total number of functional teats, and total number of inverted teats. Sex, birth herd, and birth month were included as fixed effects in our model, based on findings from a previous study (Chalkias et al. 2013). We used corrections for multiple testing and significant levels with Bonferroni correction at 1.1 × 10^−6^as the most stringent threshold and significant at a genome-wide level. Additionally, we followed the suggestions made in the paper of Teyssèdre et al. (2012) using the proposed threshold of 5 × 10^−5^ (The Wellcome Trust Case Control Consortium 2007) as highly significant, and a threshold of 5 × 10^−4^ as significant. Additional loci above a threshold of 5 × 10^−3^ were listed as suggestive.

### Further analysis of identified regions

The positions of the significantly associated SNP were assigned using the information from ENSEMBL (http://www.ensembl.org/index.html) to identify potential candidate genes. Positions and other details of previously reported QTL were downloaded from the information on the PigQTLdb (http://www.animalgenome.org/cgi-bin/gbrowse/pig/) in February 2016 (Hu et al. 2016). The positions of previously reported QTL for teat traits were compared to our findings. Previously, some of the results shown here had been combined with data from a linkage study (Jonas et al. 2008). Microsatellite markers which had not been mapped to the ENSEMBL genome in that study were further located using flanking markers according to the NCBI map-viewer (http://www.ncbi.nlm.nih.gov/projects/mapview/maps.cgi?). Haplotypes were built from the SNP data of the current study and allele frequencies compared to those of the microsatellite markers. A family-based association test was performed to identify associated regions in common between both studies and in a combined dataset (Jonas et al. 2014). Results from expression studies for the inverted teat defect were also included in the discussion of our results. The information used for the comparison is included in Supplementary material and was mostly downloaded from the PigQTLdb (http://www.animalgenome.org/cgi-bin/gbrowse/pig/) (Hu et al. 2016).

## Results and discussion

### Phenotypes

The average number of inverted teats in affected pigs in our study was 3.1, which is similar to the figures presented in earlier studies in different commercial pig lines (Beilage et al. 1996; Clayton et al. 1981; Hittel 1984; Jonas et al. 2008; Mayer and Pirchner 1995). The study of a larger Swedish Yorkshire cohort reported that in 2010, 13% of the Swedish Yorkshire animals with potential for breeding did have at least one inverted teat at the time point of selection (Chalkias et al. 2013). The number of functional teats is of importance for the early survival of the piglets. A high weaning rate requires that each piglet has access to its own teat, thus obtaining a sufficient amount of nutrients. The number of teats is therefore an important trait for efficient piglet production. Thus the selection for increased number of piglets born alive requires also a focus on increased number of teats. Selective breeding does take the number of functional teats into account, and genetic progress in recent years has been achieved, even though the estimated heritability for number of teats is low to moderate (Chalkias et al. 2013; Long et al. 2010; McKay and Rahnefeld 1990). While the number of teats in the Yorkshire population has increased, the threshold for use of the animal in breeding has been at least 14 functional teats since the 1980s (Chalkias et al. 2014). The animals (including cases and controls) in the present study had between six and 16 functional teats and between 12 and 17 teats in total. Using the threshold selection would have resulted in the exclusion of six sows without inverted teats, which had only 13 teats.

Nevertheless, despite the successes to increase the total number of teats, further research is needed to also decrease the number of non-functional teats and to investigate the connection between the two traits. In the present study, the phenotypic correlation between the number of inverted teats and total number of teats was low, but positive (data not shown). Selection for the total number of teats would therefore not necessarily increase the incidence of inverted teats; however, an early study reported a low but negative genetic correlation between the number of inverted teats and total number of teats (Hittel 1984; Von Brevern et al. 1994). Also the number of inverted teats has been reported to be negatively correlated with the number of functional teats (Von Brevern et al. 1994). We also found a moderate phenotypic correlation between total number of teats and number of functional teats; however, the design of our study was focused on animals with inverted teats and is therefore biased. A study using Swedish Yorkshire animals did also indicate that the number of functional teats accessed at different ages might not be highly correlated (Chalkias et al. 2013) and the definition of the trait is therefore important when comparing studies. The selection for the total number of teats will also increase the number of non-functional teats, but the effect on nursing behaviour has yet to be shown (Chalkias et al. 2013).

Little is known on the development of the inverted teat defect. There are only relatively few recent reports on incidences in commercial sow lines. The problems of an early determination of non-functional teats in pigs (Chalkias et al. 2013), and results from a clinical study which concluded that some of the teats scored as ‘inverted’ were indeed fully functional at the time-point of the first lactation (Chalkias et al. 2014), add further difficulties to this trait. Teats remaining inverted cannot be suckled by a piglet, and the fighting amongst the piglets struggling to achieve their feed will also increase the stress laid on the sow (Chalkias et al. 2014). This adds to the problem of selection based on the udder scoring, as sows might be excluded from the breeding schemes if teats are scored as inverted, even though all teats might be fully functional at the time of the lactation. Identifying gene loci for the inverted teat defect will offer more precise selection opportunities. Many studies have attempted to map candidate regions using QTL mapping, making the number of teats one of the most well-investigated reproductive traits, but few studies have focussed on functional teats (Hu et al. 2016). It has also been suggested that the selection for number of teats will also affect the variance of the teat number, but a selection for reduced variance of the teat number will have a limited effect as the heritability is low (Felleki and Lundeheim 2013, 2015).

### Association analysis

**Fig. 1:**
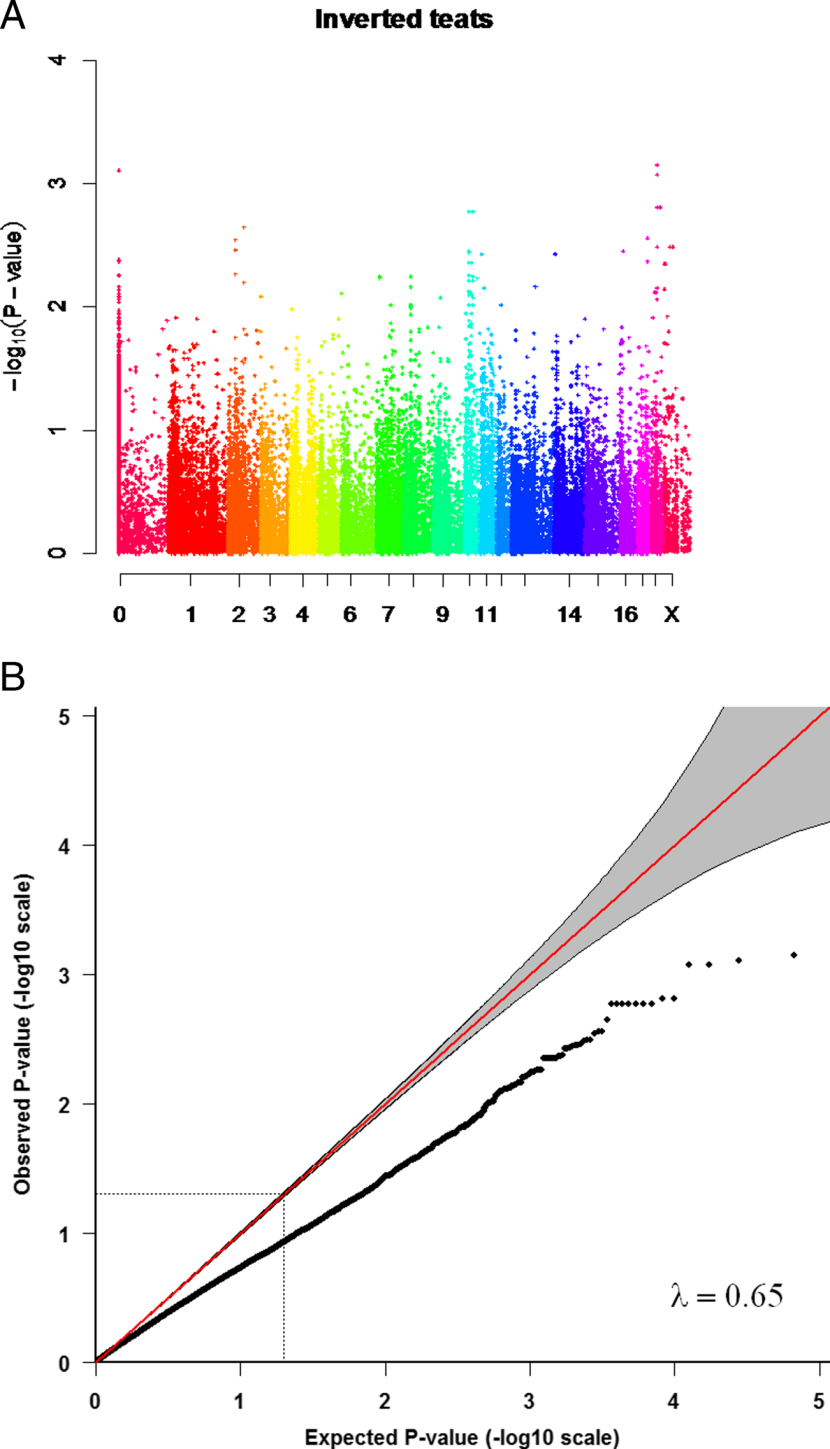
The association with the number of inverted teats and QQ-plot for the number of inverted teats. **a** Manhattan plot showing the negative logarithm of the *p*-value from the genome-wide association analysis for the number of inverted teats. Different colours represent chromosomes from 1 to X; results from unmapped markers not yet mapped to the pig reference genome are shown on the left side. **b** QQ plot from the GRAMMAR–Gamma analysis, inflation factor =0.65)

**Fig. 2:**
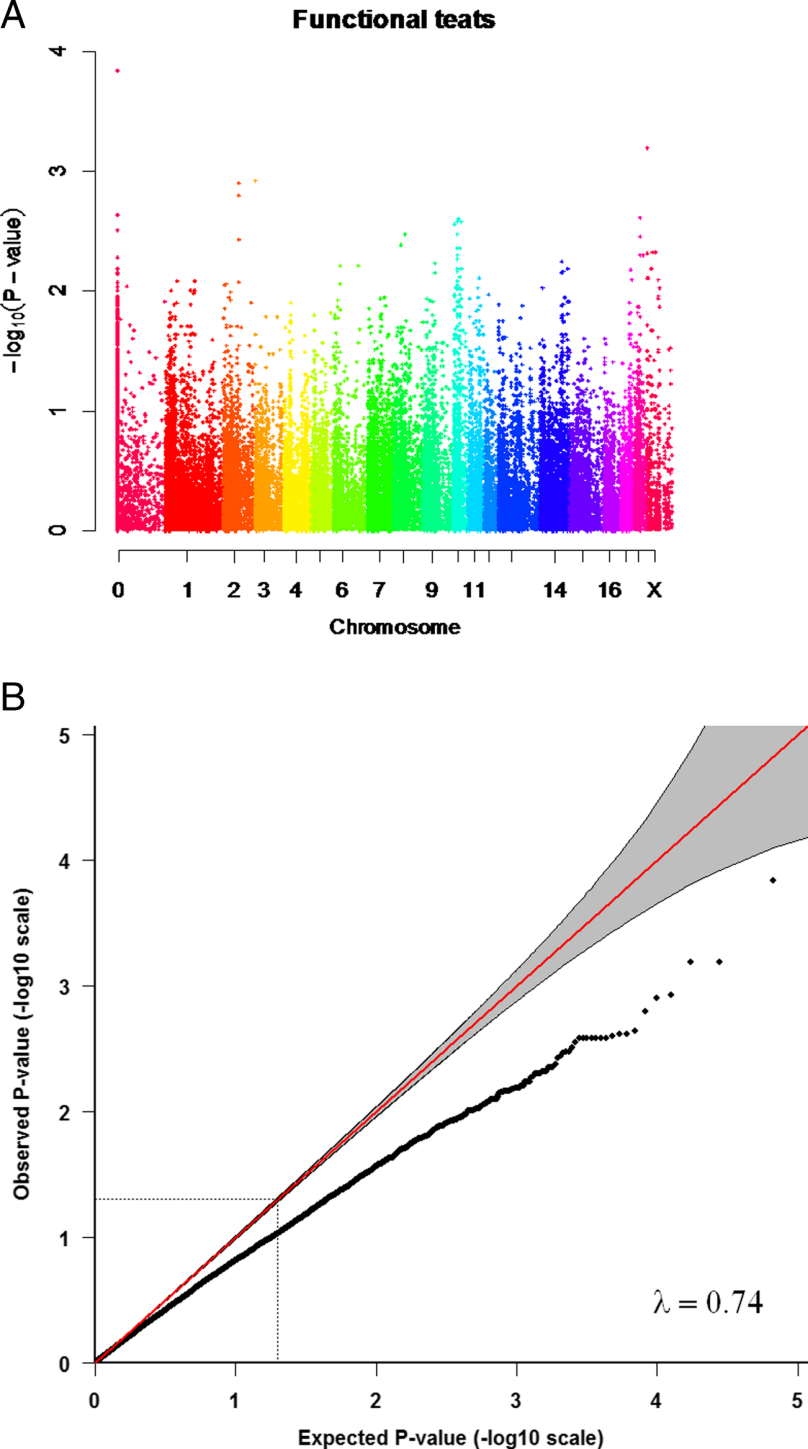
The association with the number of functional teats and QQ-plot for the number of functional teats. **a** Manhattan plot showing the negative logarithm of the *p*-value from the genome-wide association analysis for the number of functional teats. Different colours represent chromosomes from 1 to X; results from unmapped markers not yet mapped to the pig reference genome are shown on the left side. **b** QQ plot from the GRAMMAR–Gamma analysis, inflation factor =0.74

**Fig. 3:**
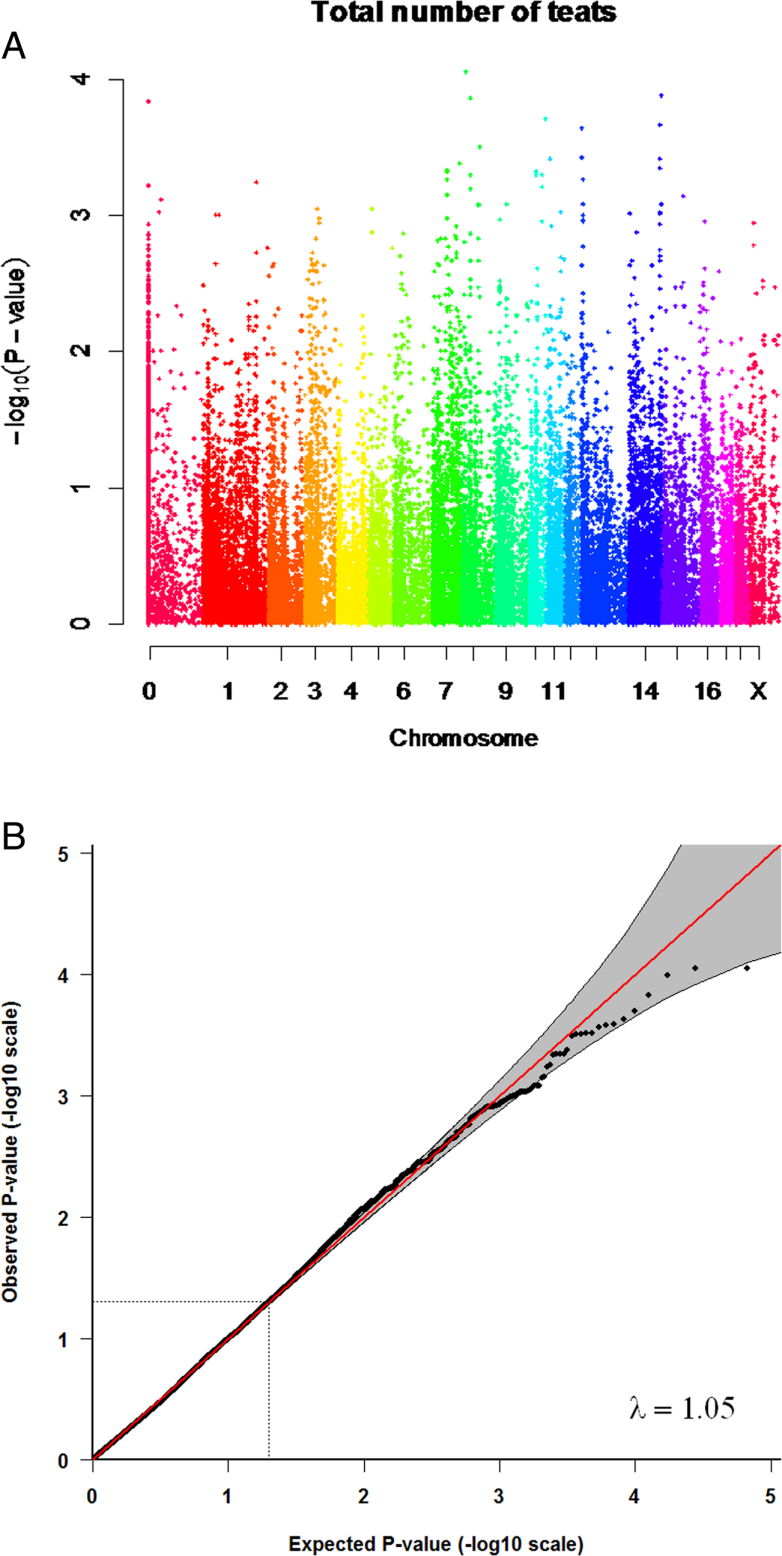
The association with total number of teats and QQ-plots for total number of teats. **a** Manhattan plot showing the negative logarithm of the p-value from the genome-wide association analysis for total number of teats. Different colours represent chromosomes from 1 to X; results from unmapped markers not yet mapped to the pig reference genome are shown on the left side. **b** QQ plot from the GRAMMAR–Gamma analysis, inflation factor =1.05

The IBS distance clustering showed limited genetic differentiation or subpopulations in the sample set. The QQ plots were deflated with an inflation factor (λ) of 0.65 (Fig. 1b) for the total number of inverted teats and λ = 0.74 for the number of functional teats (Fig. 2b). This suggests that there are external factors that add noise to the data that we could not account for. If the study design and the choice of animals based on the affected sib pair design, had led to the deflated QQ plots is unclear. The QQ plots for the total number of teats was slightly inflated, with a λ of 1.05 (Fig. 3b).

We identified regions on the genome showing significant and suggestive association with the total number of inverted teats, the number of functional teats and the total number of teats (Figs. 1a, 2a, and 3a). Positions of markers showing the lowest *p*-value for number of inverted teats and number of functional teats, as well as additional suggestive SNPs in the same regions on chromosomes 2, 10, and 18 are shown in Table 1. One unmapped marker with potential impact on the total number of inverted teats was identified. There have been only a few studies on genetic markers for the inverted teat defect or other teat abnormalities in pigs. Quantitative trait loci (QTLs) have previously been reported on different chromosomes for inverted teats (Jonas et al. 2008) and for extremely small teats (Sato et al. 2006), while expression studies were performed for the inverted teat (Chomwisarutkun et al. 2012a, b, 2013). The region identified on chromosome 2 overlapped to both the previous linkage studies (Jonas et al. 2008; Sato et al. 2006) and the expression study (Chomwisarutkun et al. 2012b). Results from a previous study did also present a suggestive QTL in a commercial pig lines for the region on chromosome 10 (Jonas et al. 2008), as most of our identified loci did also not exceed the significance threshold. While the QTL identified using pigs from an experimental population did reach the significance threshold, this QTL was in another position on the same chromosome (Jonas et al. 2008). However, gene expression differences between tissue from inverted and normal teats of affected sows and between inverted and normal teats of affected and normal sows were observed in the same regions on chromosome 10 (Chomwisarutkun et al. 2012b). The results on chromosome 18 did confirmed regions identified in previous linkage studies, while the differentially expressed genes were identified in different regions on that chromosome. The combined analysis performed earlier confirmed a significant association of a marker region on chromosome 18 around marker SW787 using the microsatellite marker or a SNP-derived haplotype in both, the pigs from the study here, and an earlier study (Jonas et al. 2014). Additional regions were identified using linkage and expression analysis in German maternal pig lines on chromosomes 1, 4 (partly overlapping regions, expression less strong), 5 (partly overlap of regions), 6 (results from expression study not very supportive), 8, 13, and 14 (Chomwisarutkun et al. 2012b; Jonas et al. 2008). The associated regions on chromosomes 3 and 11, using linkage and expression studies, did not overlap (Chomwisarutkun et al. 2012b; Jonas et al. 2008; Sato et al. 2006). These regions were not identified in the present study. Most interestingly, all identified loci associated with the number of inverted teats in our study were also associated with the number of functional teats. Both traits were highly negatively correlated in our pig material (data not shown) and these traits have previously been shown to be genetically correlated (Chalkias et al. 2013), which might explain the overlapping regions. Two associated regions, which were not associated with the total number of inverted teats but only with the number of functional teats, were identified on chromosomes 3 and X (Table 1); these loci did not exceed the significance threshold, and regions did not align with those identified in the gene expression study (Chomwisarutkun et al. 2012b).

**Table 1:** Markers showing the lowest *p*-values, their chromosome positions and their closest genes

Trait	Marker	*P*-value	^a^Sign.	^b^Chr: position (Sscrofa10.2)	Candidate gene	^d^Distance (kbp)
FT	ASGA0093674	1.45 × 10^−4^	*	2:150075004	Sprouty homolog 4	0
IT	ASGA0093674	7.80 × 10^−4^	*	2:150075004	Sprouty homolog 4	1
FT	ASGA0094376	2.30 × 10^−3^	^#^	2:150146150	Sprouty homolog 4	79
FT	MARC0054325	1.25 × 10^−3^	^#^	2:150265189	Fibroblast growth factor1	52
FT	MARC0089203	1.60 × 10^−3^	^#^	2:150320761	Fibroblast growth factor1	0
FT	H3GA0055317	1.19 × 10^−3^	^#^	3:1456146	Extracellular leucine-rich repeat and fibronectin type-III domain-containing protein 12	31
TT	ASGA0105179	1.46 × 10^−4^	*	6:16482264	Cytochrome b5 type B	0
TT	ASGA0102674	4.50 × 10^−5^	**	6:16527382	Cytochrome b5 type B	22
TT	MARC0043725	8.60 × 10^−5^	*	8:16904240	Probable G-protein coupled receptor 125	1
TT	ASGA0038917	1.36 × 10^−4^	*	8:73904815	Ras association (RalGDS/AF-6) domain family member 6	6
TT	H3GA0024944	1.36 × 10^−4^	*	8:74188735	Platelet factor 4	8
TT	DIAS0002150	3.10 × 10^−4^	*	^c^8	-	-
IT	MARC0039401	1.69 × 10^−3^	^#^	^c^10	-	-
TT	ASGA0096380	1.94 × 10^−4^	*	^c^10	-	-
IT	MARC0007270	1.69 × 10^−3^	^#^	10:37039997	ENSSSCG00000021050.1	0
IT	ALGA0058324	1.69 × 10^−3^	^#^	10:37080923	ENSSSCG00000024384.1	1
IT	H3GA0029901	1.69 × 10^−3^	^#^	10:37334494	aquaporin 3	
FT	ALGA0058701	2.50 × 10^−3^	^#^	10:46983347	Rho GTPase activating protein 121	190
TT	ALGA0067420	2.29 × 10^−4^	*	13:1776562	ENSSSCG00000011185.1	4
TT	MARC0057998	2.16 × 10^−4^	*	14:142385942	Fibroblast growth factor receptor 2	184
TT	ASGA0067411	1.31 × 10^−4^	*	14:144719642	Probable G-protein coupled receptor 125	88
IT	ALGA0098356	1.54 × 10^−3^	^#^	18:47975445	Carboxypeptidase vitellogenic-like	0
IT	ALGA0098358	7.06 × 10^−4^	*	18:48005643	Carboxypeptidase vitellogenic-like	0
IT	DRGA0017028	1.54 × 10^−3^	^#^	18:48060045	Carboxypeptidase vitellogenic-like	198
FT	ALGA0098367	2.41 × 10^−3^	^#^	18:48114245	Carboxypeptidase vitellogenic-like	54
IT	ALGA0098367	8.44 × 10^−4^	^#^	18:48114245	Carboxypeptidase vitellogenic-like	54
FT	DRGA0017032	2.41 × 10^−3^	^#^	18:48127365	Carboxypeptidase vitellogenic-like	67
IT	DRGA0017032	8.44 × 10^−4^	^#^	18:48127365	Carboxypeptidase vitellogenic-like	67
FT	ASGA0080792	6.41 × 10^−4^	^#^	X:10700485	CH242-336 J8.1	1
FT	H3GA0051511	6.41 × 10^−4^	^#^	X:10712229	CH242-336 J8.1	11

IT: number of inverted teats; FT: number of functional teats; TT: total number of teats

^a^Significance threshold (***significant at genome-wide level using Bonferroni correction (1.1 × 10^−6^); **highly significant (5 × 10^−5^); *significant (5 × 10^−4^); ^#^suggestive (5 × 10^−3^))

^b^Chromosome position in ENSEMBL

^c^Variation does not map to the genome or no hit

^d^Distance from nearest gene

The identified loci associated with the number of inverted and functional teats were distinct from those identified for the total number of teats. QTLs were identified on chromosome 6, 8, 13, and 14 (Table 1). One additional marker was significantly associated with the total number of teats and was located on chromosome 10; however, this marker has not been mapped on the latest ENSEMBL assembly. QTLs for the number of teats have previously been reported across all autosomes and a total of 167 QTLs for the total number of teats, ten QTLs for the number of teats on the left side and seven QTLs for the number of teats on the right side have been assembled on the PigQTL database (Hu et al. 2013, 2016). Especially recent genome-wide association studies have added more significant markers to the database (Arakawa et al. 2015; Duijvesteijn et al. 2014; Hernandez et al. 2014; Verardo et al. 2014). We extracted the information on traits and QTL positions from the PigQTL database to compare the results with our findings (Supplementary files). We did not identify any significant association on chromosomes 1, 3, 4, 7, 12, or 15, the chromosomes with most reported QTLs for the number of teats. However, the QTLs we identified on chromosomes 6, 8, and 10 were among those more frequently reported in the PigQTL database (Hu et al. 2016). The QTL on chromosome 6 aligned well with the identified regions in two previous studies (Guo et al. 2008; Hernandez et al. 2014), but the results were distinct from those in six other studies (Arakawa et al. 2015; Cassady et al. 2001; Ding et al. 2009; Holl et al. 2004; Tortereau et al. 2010; Zhang et al. 2007). We identified markers in two regions on chromosome 8, from which one region aligned well with those reported in recent studies (Duijvesteijn et al. 2014; Verardo et al. 2014) and the other was also overlapping with results from linkage analysis studies (Beeckmann et al. 2003; Cassady et al. 2001; Duijvesteijn et al. 2014; King et al. 2003; Sato et al. 2006). Two recent studies have identified a total of three QTLs on chromosome 14, where we identified one of the most associated markers (Arakawa et al. 2015; Duijvesteijn et al. 2014), and one study also reported one significant marker on chromosome 13 (Arakawa et al. 2015); however, the positions did not overlap, with marker ALGA0067420 at 1,776,562 bp and marker ALGA0071614 at 108,361,945 bp (http://www.animalgenome.org/repository/pig/Genome_10.2_mappings/SNP_pos_build10.2_final.txt). Results of the combined assembly are shown in Supplemental file X.

Despite being able to identify associated markers, there was a lack of strong associations: one of the suggestions is that animals from the Yorkshire population have been highly selected for maternal traits, including number of teats. The observed phenotypes did verify this, as no animal with an extreme high or low number of teats was found. A QTL verification study using commercial sow lines did identify similar difficulties when comparing results from a cross between extreme breeds and such from commercial maternal lines (Jonas et al. 2008). A combined analysis with these results had been presented earlier, and it was suggested that the definition of the phenotype is relevant but also differences of the population and the genetic background of the trait might exist (Jonas et al. 2014).

### Candidate genes

Even though candidate genes have been identified, no major gene has been reported for the number of teats or especially, functional teats in pigs. Previous studies have suggested a number of candidate genes for the inverted teats defect derived from the findings in linkage and expression studies (Chomdej 2005; Trakooljul 2004; Yammuen-Art 2008). Candidate gene studies identified significant associations with the relaxin gene (*RLN*) on chromosome 1, the parathyroid hormone-like hormone gene (*PTHLH*) on chromosome 5, and the transforming growth factor beta 1 gene (*TGFB1*) gene on chromosome 6 in an experimental population (Chomdej 2005). The association of *PTHLH* was also confirmed in commercial pig lines (Martinez-Giner et al. 2011; Tetzlaff et al. 2009), but none of the markers in our study showed significant association with the inverted teat defect on chromosomes 1, 6, or 5. An expression study based on results from a microarray comparing tissues from inverted and normal teats from sows with and without inverted teats aimed to validate the differential expression of five candidate genes. The connective tissue growth factor (*CTGF*) on chromosome 1 was found to be highly expressed in inverted teats, while the growth differentiation factor 8 (*GDF8*) on chromosome 15 was highly expressed in normal teats. The differential expression of the epidermal growth factor (*EGF*) on chromosome 8 and its receptor (*EGFR*) on chromosome 9 did not align to the significant regions in our study (Yammuen-Art 2008). The same study identified additional candidate genes from the literature, and found that the G-protein coupled receptor 135 (*GPCR135*) was more highly expressed in teats from affected sows than in normal sows (Yammuen-Art 2008). However, none of those genes were located on chromosomes identified for the inverted teat defect in our study.

The expression study also suggested relaxin 3 (*RLN3*) on chromosome 2 as a potential candidate gene, and it was found to be more highly expressed in teats from affected sows than in normal sows, but this gene was not associated with the inverted teat defect (Yammuen-Art 2008). Another gene on chromosome 2, insulin-like growth factor 2 (*IGF-II*), was also found to be highly expressed in inverted teats in the same study (Yammuen-Art 2008). These genes did not align with the positions of the suggestive and significantly associated markers in our study. We identified other potential candidate genes, including the fibroblast growth factor 1 (*FGF1*), which is supposed to be involved in promoting mammary ductal development during sexual maturity in mice (Akers 2006). The marker ASGA0093674 is located within the sprout homolog 4 (*SPRY4*) gene (Table 1). *SPRY4* is an inhibitor of the mitogen-activated protein kinase (*MAPK*) and suppresses the insulin receptor. However, there is no clear suggestion on how this impacts the development of the teat. Other genes previously suggested as potential candidate genes for the inverted teat defect on chromosome 2 are the follicle-stimulating hormone (*FSH*) and peptidylprolyl cis/trans isomerase (*PIN1*) (Jonas 2007); however, both are not present in our candidate regions.

The associated marker MARC0039401 had been in the initial mapping information located on chromosome 10, but has not been assigned to a position in the most recent ENSEMBL database or on the animal genome (http://www.animalgenome.org/repository/pig/Genome_10.2_mappings/SNP_pos_build10.2_final.txt) repository from the build 10.2. The gene aquaporin 3 (*AQP3*) is a potential candidate gene on this chromosome as it is a glycerol transporter in the mammalian skin (Hara-Chikuma and Verkman 2005).

We identified on chromosome 18, together with chromosome 2, the markers most associated with the number of inverted and functional teats. The markers with the lowest *p*-values (ALGA0098367 and DRGA0017032) are located 13 kb from each other and about 60 kb from the carboxypeptidase vitellogenic-like (*CPVL*) gene on chromosome 18. The protein encoded by this gene is a carboxypeptidase; however, the exact function of this protein has not been determined.

Significant and suggestive associations were identified for the number of functional teats, but not for the inverted teats on chromosomes 13 and 14 and the X chromosome. The marker MARC0057998 is located 184 kb from fibroblast growth factor receptor 2 (*FGF2*) on chromosome 14. The signalling of the FGF family is relevant for mammary gland development, and it has been shown that *FGF2* controls the ductal elongation (Zhang et al. 2014). The androgen receptor (*AR*) on the X chromosome had previously been proposed as a potential candidate gene for the inverted teat defect (Trakooljul 2004); however, this gene does not align to the position of the significant markers identified in our study.

Some of the candidate genes previously identified are located on chromosomes on which we identified significantly associated markers for the total number of teats. The *TGFB1* gene on chromosome 6 (Chomdej 2005) did not align to the most significant markers identified for the total number of teats in our study, or that previously reported by Cassady et al. (2001). The cytochrome B5 type B (*CYB5B*) gene is located closely to the associated marker on chromosome 6, but we could not find a study clearly suggesting its role in the development of the mammary tissue. The other interesting chromosome for the total number of teats is chromosome 8, where Bidanel et al. (2008) also detected a number of QTLs for number of teats. Mendez et al. (1999) did map *EGF* to this chromosome, a gene which showed differential expression in a microarray study (Yammuen-Art 2008). Again, our most highly associated SNPs did not map to the region of this gene.

In summary, we could not confirm the positions of previously identified and suggested genes for the inverted teat defect, but this study suggests a number of novel potential candidate genes for this trait. The reasons for lack of confirmation in our study might be several and it is hypothesized that other factors, including regulatory elements, influence teat development. Further, the comparison in the mRNA expression profiles of teat tissues (normal and inverted) from pigs with and without inverted teats did not lead to any conclusive knowledge of the genetic control of this inherited defect (Chomwisarutkun et al. 2012a).

## Conclusion

This study suggests new candidate regions and genes for the teat traits investigated, especially the inverted teat defect for which only few studies have been published so far. Our results suggest that some regions, especially on chromosomes 2 and 18, might harbour favourable alleles, but this needs to be further investigated in more pigs. Two candidate genes, *SPRY4* and *FGF1*, were identified on chromosome 2 and one candidate gene, *CPVL*, in close approximation with the five significant markers on chromosome 18. We were able to confirm that regions on chromosomes 2, 8, 10, and 18 are relevant for the inverted teat defect; these chromosomes had been previously identified in linkage and expression studies. However, the regions for the identification of potential candidate genes did differ.

Our study did confirm some of the previous results, and we conclude that the total number, the number of functional, and the number of inverted teats are traits with a complex regulative pathway. Further studies and a better understanding of the development and the biology underlying the inverted teat defect are needed. This will especially assist future breeding decisions to ensure that the early selection for non-functional teats will improve mothering ability during lactation.

## Electronic supplementary material

Below is the link to the electronic supplementary material.

ESM 1 (PDF 1004 kb)
