# Supplementary material for: Identification of novel candidate genes for the inverted teat defect in sows using a genome-wide marker panel
Source: J Appl Genet. 2017 Jan 4;58(2):249–59. doi: 10.1007/s13353-016-0382-1 (PMC5391382; doi:10.1007/s13353-016-0382-1)
Supplement: Supplementary file 1 — (PDF 1004 kb) [file 13353_2016_382_MOESM1_ESM.pdf]

Supplementary files

Figures showing the assembly of own results and published results across all autosomes (chromosome 1-18, SSC1-18) and the X chromosome.

Shown are results for expression studies for inverted teats (green), QTL studies for inverted/ functional teats (red), association studies for inverted/ functional teats (dark red), QTL studies for number of teats (blue) as well as association studies for number of teats (black). If multiple loci were reported from the same study, loci were combined; the number of combined loci is shown in parenthesis behind the reference. It is indicated if results were significant (**, P>0.05), suggestive (*, P>0.1), or if no information given. Positions are given in approximations and information were taken from the literature and the Pig QTLDB (*Hu Z-L, Park CA, Reecy JM (2016) Developmental progress and current status of the Animal QTLdb Nucleic Acids Research 44:D827-D833 doi:10.1093/nar/gkv1233*). Charts were designed using MapChart (*Voorrips RE (2002) MapChart: Software for the Graphical Presentation of Linkage Maps and QTLs Journal of Heredity 93:77-78 doi:10.1093/jhered/93.1.77*). References used are the following:

Arakawa A et al. (2015) Genome-wide association QTL mapping for teat number in a purebred population of Duroc pigs Animal Genetics 46:571-575 doi:10.1111/age.12331

Beeckmann P, Moser G, Bartenschlager H, Reiner G, Geldermann H (2003) Linkage and QTL mapping for Sus scrofa chromosome 8 J Anim Breed Genet 120 doi:10.1046/j.0931-2668.2003.00425.x

Bidanel JP et al. (2008) Detection of quantitative trait loci for teat number and female reproductive traits in Meishan X Large White F2 pigs Animal 2:813-820 doi:10.1017/s1751731108002097

Cassady JP, Johnson RK, Pomp D, Rohrer GA, Van Vleck LD, Spiegel EK, Gilson KM (2001) Identification of quantitative trait loci affecting reproduction in pigs Journal of Animal Science 79:623-633

Cepica S, Reiner G, Bartenschlager H, Moser G, Geldermann H (2003) Linkage and QTL mapping for Sus scrofa chromosome X J Anim Breed Genet 120 doi:10.1046/j.0931-2668.2003.00436.x

Chomwisarutkun K, Murani E, Ponsuksili S, Wimmers K (2012) Gene expression analysis of mammary tissue during fetal bud formation and growth in two pig breeds - indications of prenatal initiation of postnatal phenotypic differences Bmc Developmental Biology 12 doi:10.1186/1471-213x-12-13

Ding N et al. (2009) Genome-wide QTL mapping for three traits related to teat number in a White Duroc x Erhualian pig resource population BMC Genet 10 doi:10.1186/1471-2156-10-6

Dragos-Wendrich M, Moser G, Bartenschlager H, Reiner G, Geldermann H (2003) Linkage and QTL mapping for Sus scrofa chromosome 10 J Anim Breed Genet 120 doi:10.1046/j.0931-2668.2003.00427.x

Duijvesteijn N, Veltmaat JM, Knol EF, Harlizius B (2014) High-resolution association mapping of number of teats in pigs reveals regions controlling vertebral development BMC Genomics 15:1-12 doi:10.1186/1471-2164-15-542

Geldermann H et al. (2003) Genome-wide linkage and QTL mapping in porcine F2 families generated from Pietrain, Meishan and Wild Boar crosses

Genomweite Kopplungs- und QTL-Kartierung in porcinen F2-Familien, erzeugt aus Kreuzungen von Pietrain, Meishan und Wildschwein Journal of Animal Breeding and Genetics 120:363-393 doi:10.1046/j.0931-2668.2003.00408.x

Guo YM, Lee GJ, Archibald AL, Haley CS (2008) Quantitative trait loci for production traits in pigs: a combined analysis of two Meishan x Large White populations Anim Genet 39 doi:10.1111/j.1365-2052.2008.01756.x

Hernandez SC, Finlayson HA, Ashworth CJ, Haley CS, Archibald AL (2014) A genome-wide linkage analysis for reproductive traits in F(2) Large White × Meishan cross gilts Animal Genetics 45:191-197 doi:10.1111/age.12123

Hirooka H et al. (2001) A whole-genome scan for quantitative trait loci affecting teat number in pigs J Anim Sci 79
Holl JW, Cassady JP, Pomp D, Johnson RK (2004) A genome scan for quantitative trait loci and imprinted regions affecting reproduction in pigs J Anim Sci 82
Jonas E et al. (2008) QTL for the heritable inverted teat defect in pigs Mammalian Genome 19:127-138 doi:10.1007/s00335-007-9086-5
King AH, Jiang Z, Gibson JP, Haley CS, Archibald AL (2003) Mapping quantitative trait loci affecting female reproductive traits on porcine chromosome 8 Biol Reprod 68 doi:10.1095/biolreprod.102.012955
Lee SS et al. (2003) Linkage and QTL mapping for Sus scrofa chromosome 2 J Anim Breed Genet 120 doi:10.1046/j.0931-2668.2003.00419.x
Rodriguez C et al. (2005) QTL mapping for teat number in an Iberian x Meishan pig intercross Anim Genet 36
Rohrer GA (2000) Identification of quantitative trait loci affecting birth characters and accumulation of backfat and weight in a Meishan-White Composite resource population J Anim Sci 78
Sato S et al. (2006) Identification of quantitative trait loci affecting corpora lutea and number of teats in a Meishan x Duroc F-2 resource population Journal of Animal Science 84:2895-2901 doi:10.2527/jas.2006-176
Tortereau F, Gilbert H, Heuven HCM, Bidanel J-P, Groenen MAM, Riquet J (2010) Combining two Meishan F2 crosses improves the detection of QTL on pig chromosomes 2, 4 and 6 Genetics Selection Evolution 42 doi:10.1186/1297-9686-42-42
Verardo LL, Silva FF, Varona L, Resende MDV, Bastiaansen JWM, Lopes PS, Guimarães SEF (2014) Bayesian GWAS and network analysis revealed new candidate genes for number of teats in pigs Journal of Applied Genetics 56:123-132 doi:10.1007/s13353-014-0240-y
Wada Y et al. (2000) Quantitative trait loci (QTL) analysis in a Meishan×Göttingen cross population Animal Genetics 31:376-384 doi:10.1046/j.1365-2052.2000.00696.x
Yue G, Schröffel J, Moser G, Bartenschlager H, Reiner G, Geldermann H (2003) Linkage and QTL mapping for Sus scrofa chromosome 12 J Anim Breed Genet 120 doi:10.1046/j.0931-2668.2003.00429.x
Zhang J et al. (2007) Detection of quantitative trait loci associated with several internal organ traits and teat number trait in a pig population J Genet Genomics 34 doi:10.1016/s1673-8527(07)60032-0

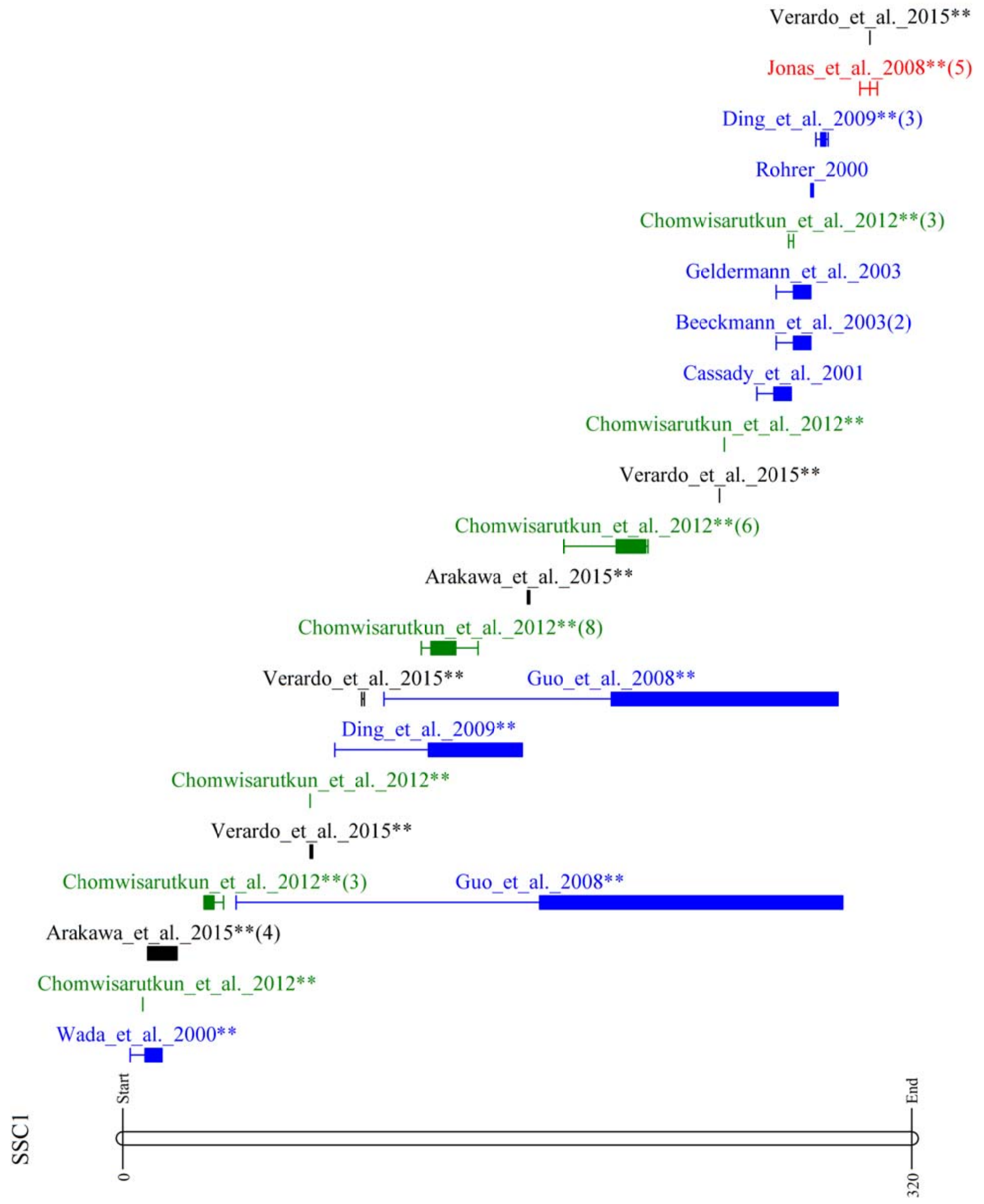
SSC1
0
Start
320
End
Wada_et_al._2000**
Chomwisarutkun_et_al._2012**
Arakawa_et_al._2015**(4)
Chomwisarutkun_et_al._2012**(3)
Guo_et_al._2008**
Verardo_et_al._2015**
Chomwisarutkun_et_al._2012**
Ding_et_al._2009**
Verardo_et_al._2015**
Guo_et_al._2008**
Chomwisarutkun_et_al._2012**(8)
Arakawa_et_al._2015**
Chomwisarutkun_et_al._2012**(6)
Verardo_et_al._2015**
Chomwisarutkun_et_al._2012**
Cassady_et_al._2001
Beeckmann_et_al._2003(2)
Geldermann_et_al._2003
Chomwisarutkun_et_al._2012**(3)
Rohrer_2000
Ding_et_al._2009**(3)
Jonas_et_al._2008**(5)
Verardo_et_al._2015**

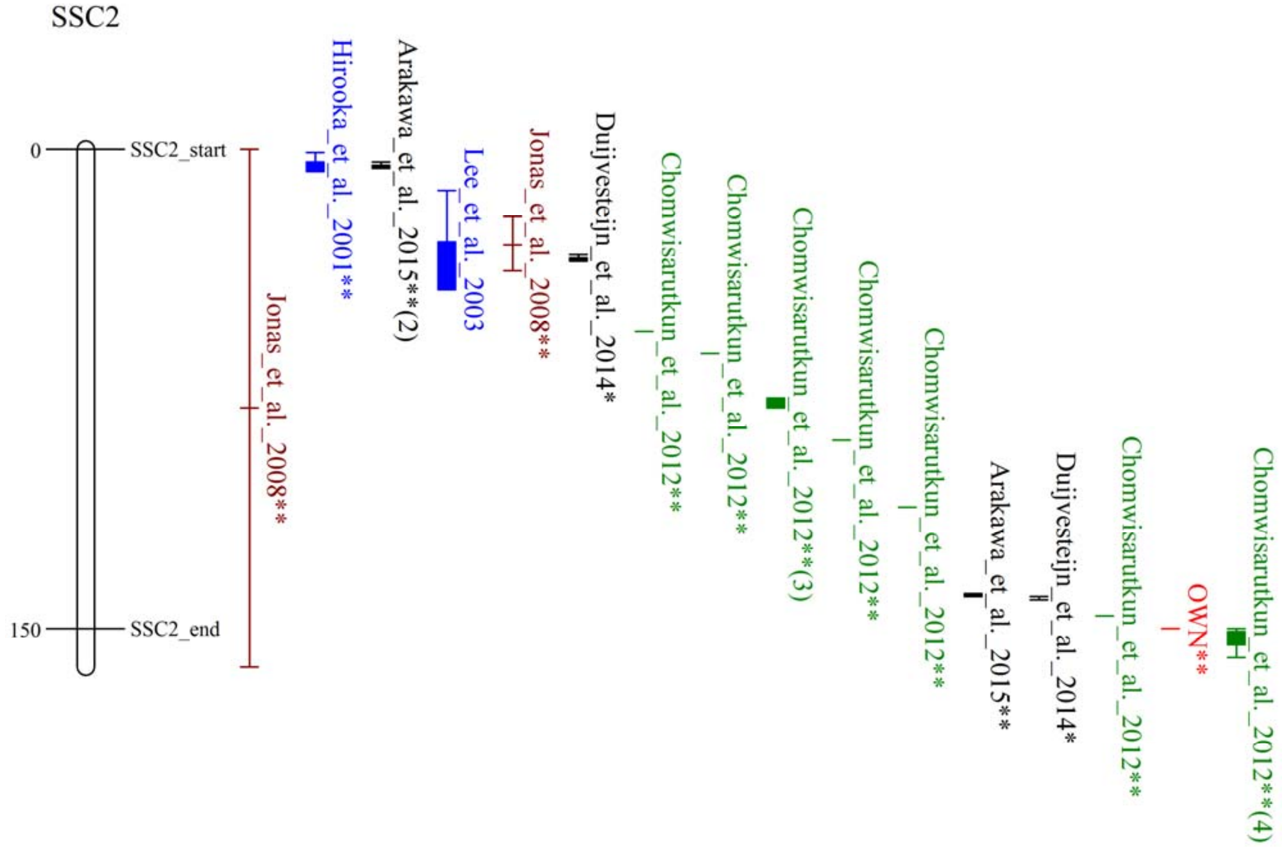
SSC2
0
SSC2_start
150
SSC2_end
Jonas_et_al._2008**
Hirooka_et_al._2001**
Arakawa_et_al._2015**(2)
Lee_et_al._2003
Jonas_et_al._2008**
Duijvesteijn_et_al._2014*
Chomwisarutkun_et_al._2012**
Chomwisarutkun_et_al._2012**
Chomwisarutkun_et_al._2012**(3)
Chomwisarutkun_et_al._2012**
Chomwisarutkun_et_al._2012**
Arakawa_et_al._2015**
Duijvesteijn_et_al._2014*
Chomwisarutkun_et_al._2012**
OWN**
Chomwisarutkun_et_al._2012**(4)

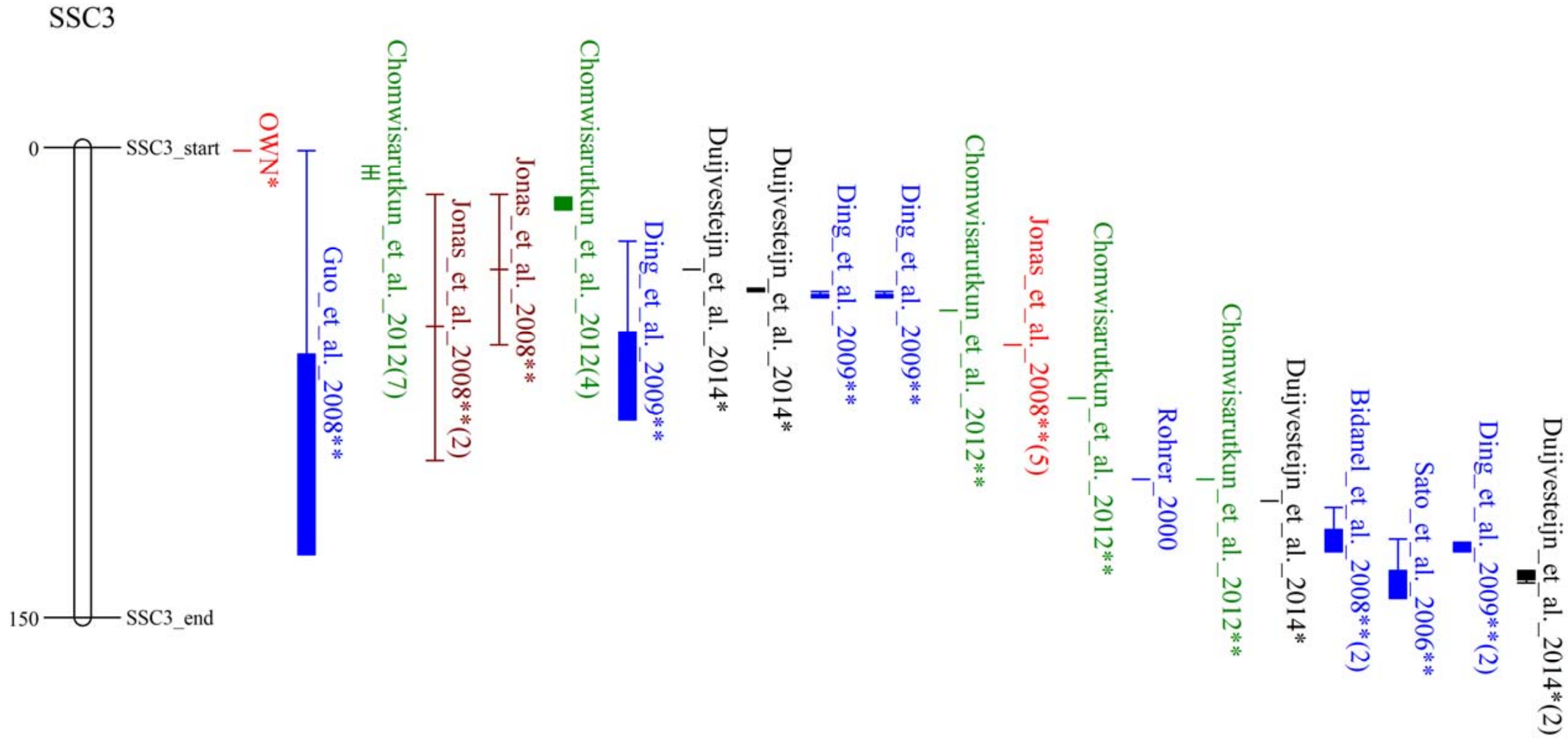

SSC3
0
150
SSC3_start
SSC3_end
OWN*
Guo_et_al._2008**
Chomwisarutkun_et_al._2012(7)
Jonas_et_al._2008**(2)
Jonas_et_al._2008**
Chomwisarutkun_et_al._2012(4)
Ding_et_al._2009**
Duijvesteijn_et_al._2014*
Duijvesteijn_et_al._2014*
Ding_et_al._2009**
Ding_et_al._2009**
Chomwisarutkun_et_al._2012**
Jonas_et_al._2008**(5)
Chomwisarutkun_et_al._2012**
Rohrer_2000
Chomwisarutkun_et_al._2012**
Duijvesteijn_et_al._2014*
Bidanel_et_al._2008**(2)
Sato_et_al._2006**
Ding_et_al._2009**(2)
Duijvesteijn_et_al._2014*(2)

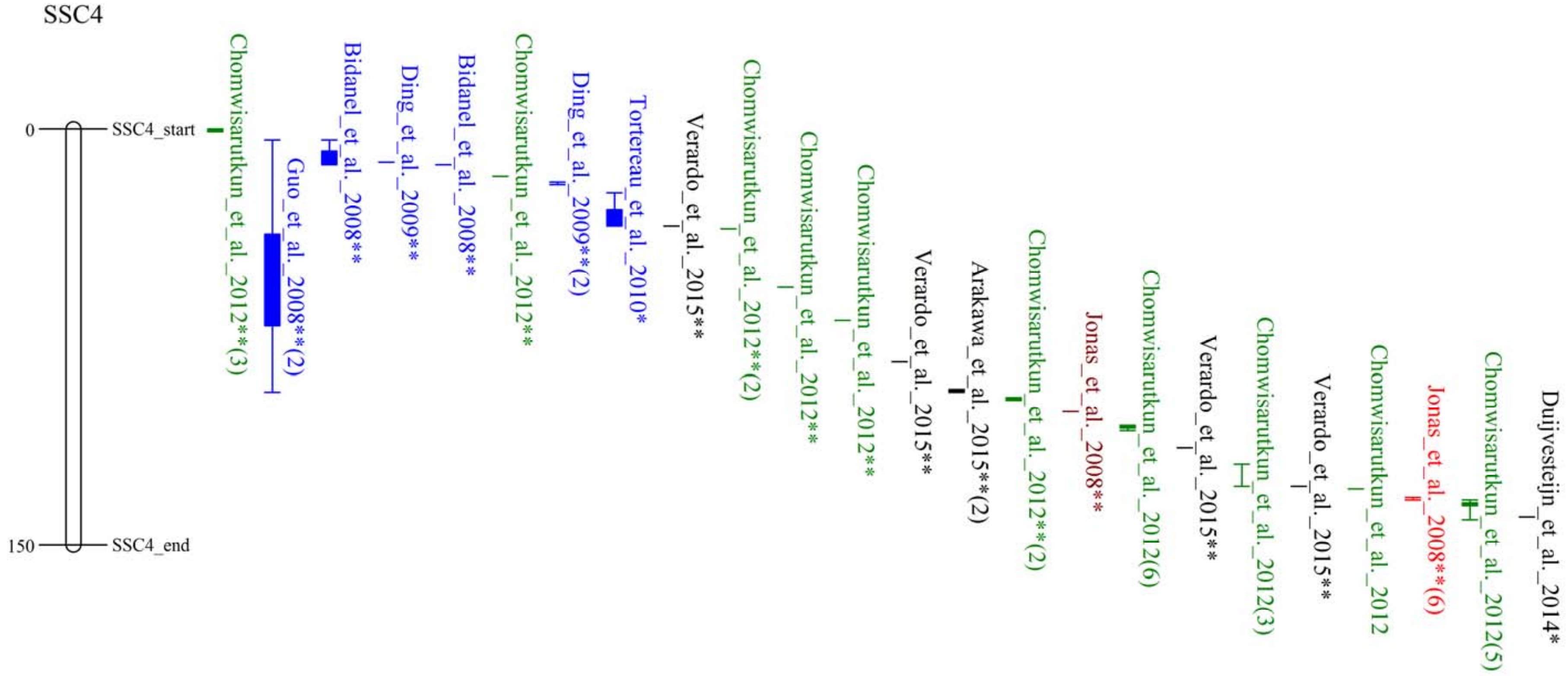

SSC4
0
150
SSC4_start
SSC4_end
Chomwisarutkun_et_al._2012**(3)
Guo_et_al._2008**(2)
Bidanel_et_al._2008**
Ding_et_al._2009**
Bidanel_et_al._2008**
Chomwisarutkun_et_al._2012**
Ding_et_al._2009**(2)
Tortereau_et_al._2010*
Verardo_et_al._2015**
Chomwisarutkun_et_al._2012**(2)
Chomwisarutkun_et_al._2012**
Chomwisarutkun_et_al._2012**
Verardo_et_al._2015**
Arakawa_et_al._2015**(2)
Chomwisarutkun_et_al._2012**(2)
Jonas_et_al._2008**
Chomwisarutkun_et_al._2012(6)
Verardo_et_al._2015**
Chomwisarutkun_et_al._2012(3)
Verardo_et_al._2015**
Chomwisarutkun_et_al._2012
Jonas_et_al._2008**(6)
Chomwisarutkun_et_al._2012(5)
Duijvesteijn_et_al._2014*

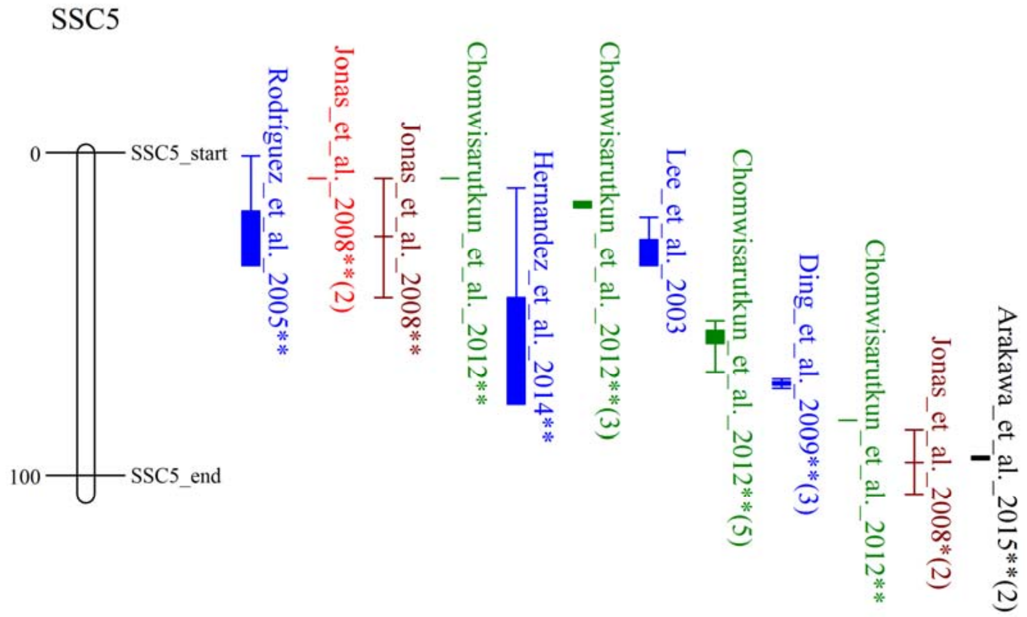
SSC5
0
100
SSC5_start
SSC5_end
Rodríguez_et_al._2005**
Jonas_et_al._2008**(2)
Jonas_et_al._2008**
Chomwisarutkun_et_al._2012**
Hernandez_et_al._2014**
Chomwisarutkun_et_al._2012**(3)
Lee_et_al._2003
Chomwisarutkun_et_al._2012**(5)
Ding_et_al._2009**(3)
Chomwisarutkun_et_al._2012**
Jonas_et_al._2008*(2)
Arakawa_et_al._2015**(2)

SSC6

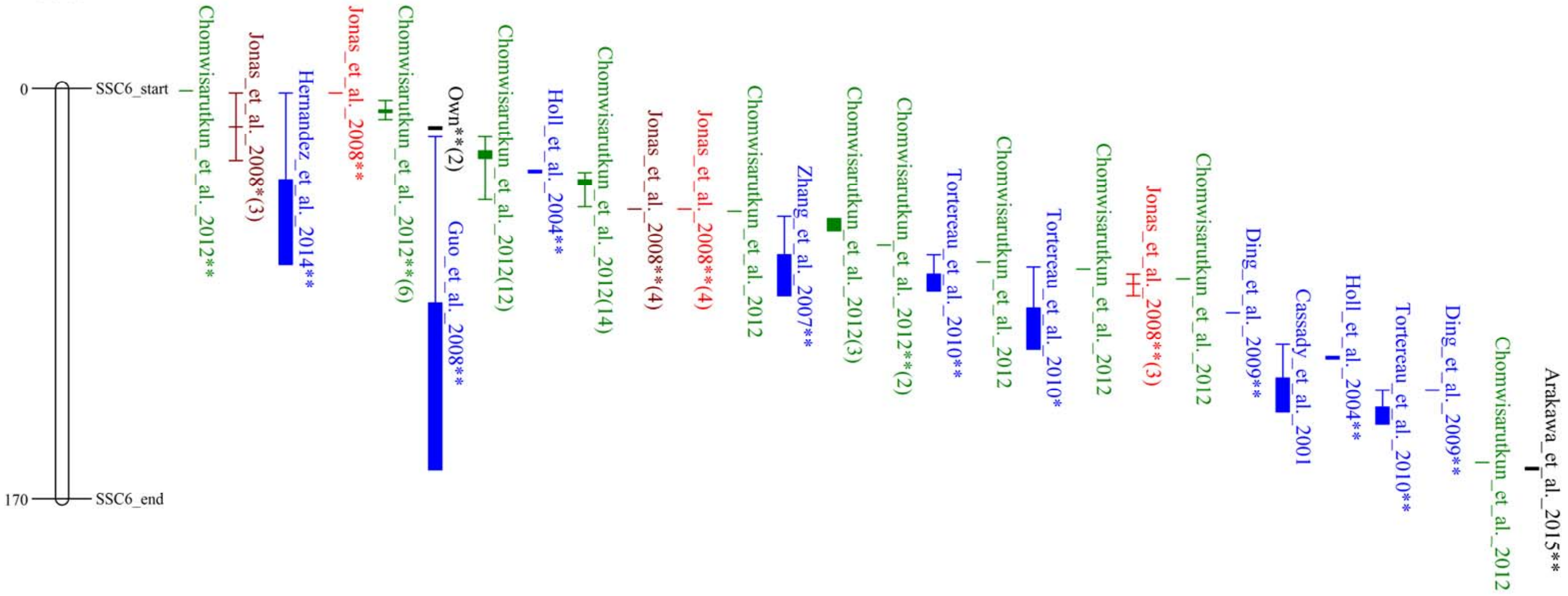
0
SSC6_start
170
SSC6_end
Chomwisarutkun_et_al._2012**
Jonas_et_al._2008*(3)
Hernandez_et_al._2014**
Jonas_et_al._2008**
Chomwisarutkun_et_al._2012**(6)
Own**(2)
Guo_et_al._2008**
Chomwisarutkun_et_al._2012(12)
Holl_et_al._2004**
Chomwisarutkun_et_al._2012(14)
Jonas_et_al._2008**(4)
Jonas_et_al._2008**(4)
Chomwisarutkun_et_al._2012
Zhang_et_al._2007**
Chomwisarutkun_et_al._2012(3)
Chomwisarutkun_et_al._2012**(2)
Tortereau_et_al._2010**
Chomwisarutkun_et_al._2012
Tortereau_et_al._2010*
Chomwisarutkun_et_al._2012
Jonas_et_al._2008**(3)
Chomwisarutkun_et_al._2012
Ding_et_al._2009**
Cassady_et_al._2001
Holl_et_al._2004**
Tortereau_et_al._2010**
Ding_et_al._2009**
Chomwisarutkun_et_al._2012
Arakawa_et_al._2015**

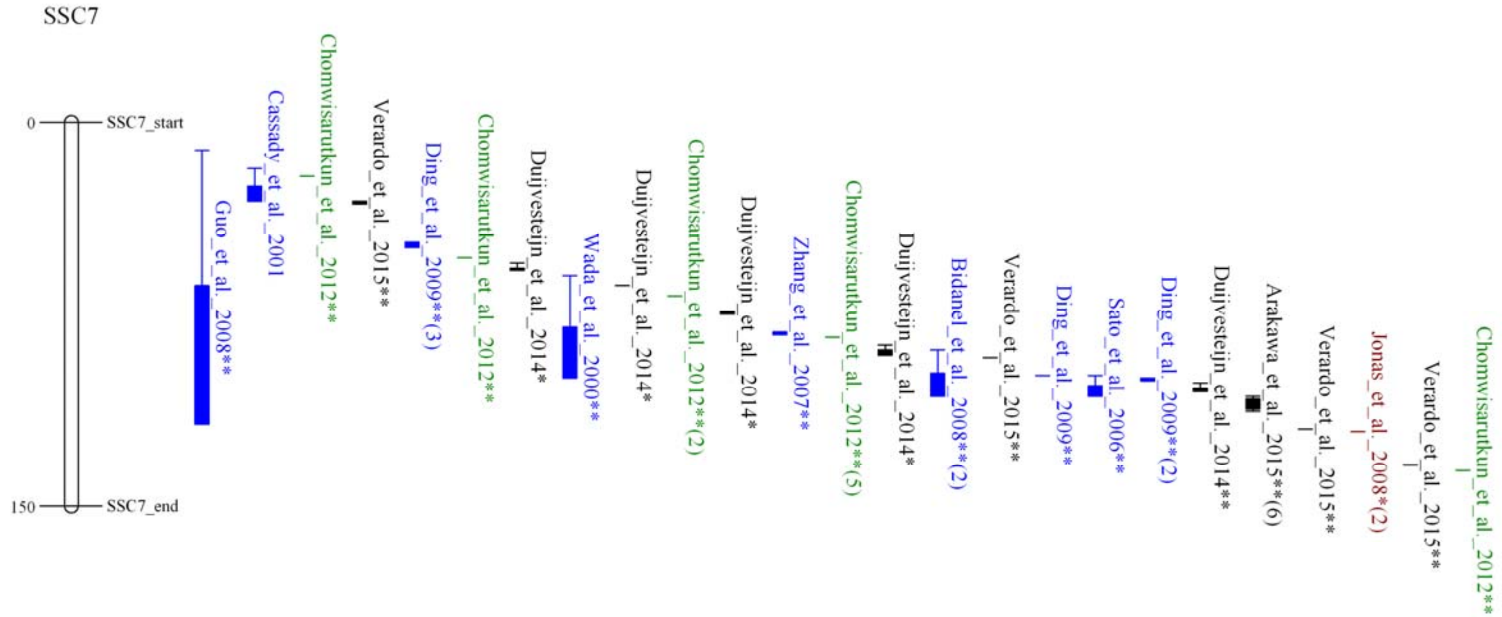
SSC7
0
SSC7_start
150
SSC7_end
Guo_et_al._2008**
Cassady_et_al._2001
Chomwisarutkun_et_al._2012**
Verardo_et_al._2015**
Ding_et_al._2009**(3)
Chomwisarutkun_et_al._2012**
Duijvesteijn_et_al._2014*
Wada_et_al._2000**
Duijvesteijn_et_al._2014*
Chomwisarutkun_et_al._2012**(2)
Duijvesteijn_et_al._2014*
Zhang_et_al._2007**
Chomwisarutkun_et_al._2012**(5)
Duijvesteijn_et_al._2014*
Bidanel_et_al._2008**(2)
Verardo_et_al._2015**
Ding_et_al._2009**
Sato_et_al._2006**
Ding_et_al._2009**(2)
Duijvesteijn_et_al._2014**
Arakawa_et_al._2015**(6)
Verardo_et_al._2015**
Jonas_et_al._2008*(2)
Verardo_et_al._2015**
Chomwisarutkun_et_al._2012**

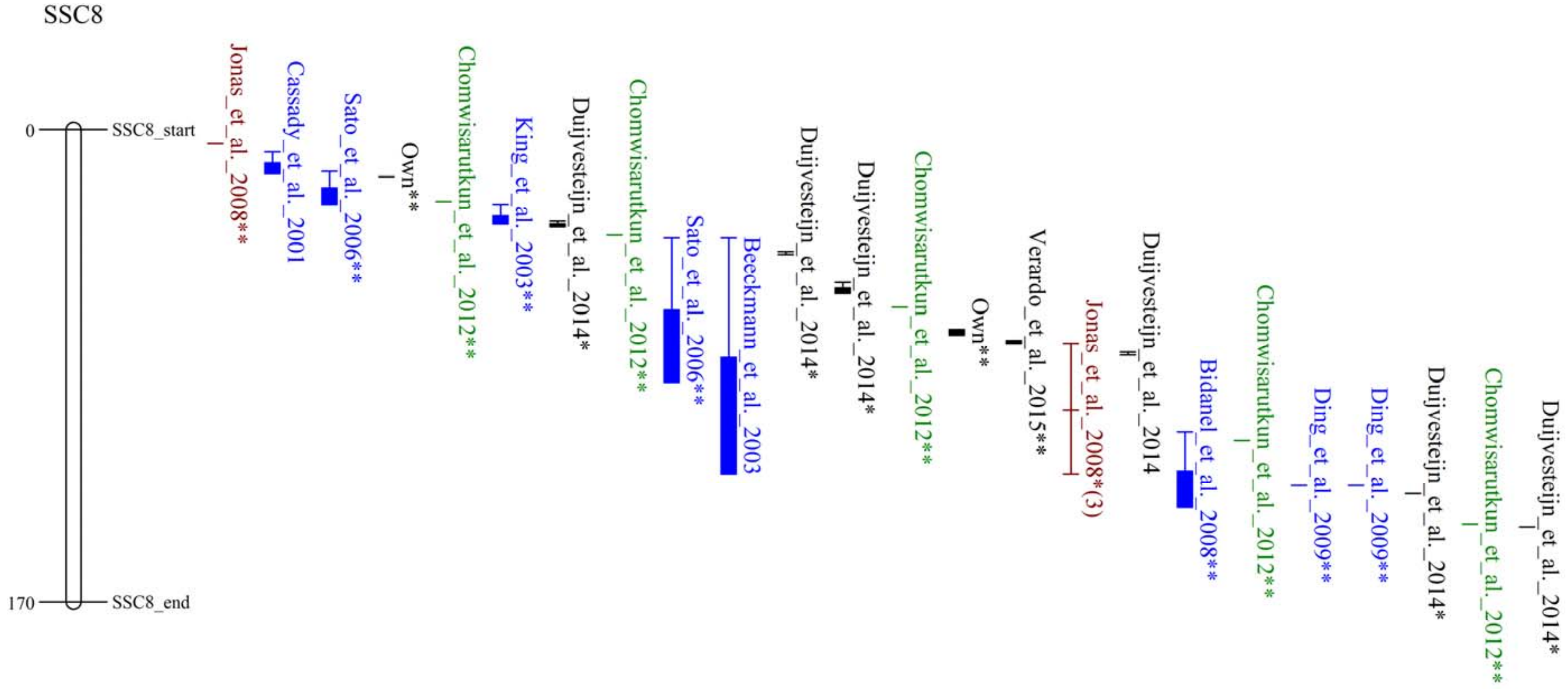

SSC8
0
SSC8_start
170
SSC8_end
Jonas_et_al._2008**
Cassady_et_al._2001
Sato_et_al._2006**
Own**
Chomwisarutkun_et_al._2012**
King_et_al._2003**
Duijvesteijn_et_al._2014*
Chomwisarutkun_et_al._2012**
Sato_et_al._2006**
Beeckmann_et_al._2003
Duijvesteijn_et_al._2014*
Duijvesteijn_et_al._2014*
Chomwisarutkun_et_al._2012**
Own**
Verardo_et_al._2015**
Jonas_et_al._2008*(3)
Duijvesteijn_et_al._2014
Bidanel_et_al._2008**
Chomwisarutkun_et_al._2012**
Ding_et_al._2009**
Ding_et_al._2009**
Duijvesteijn_et_al._2014*
Chomwisarutkun_et_al._2012**
Duijvesteijn_et_al._2014*

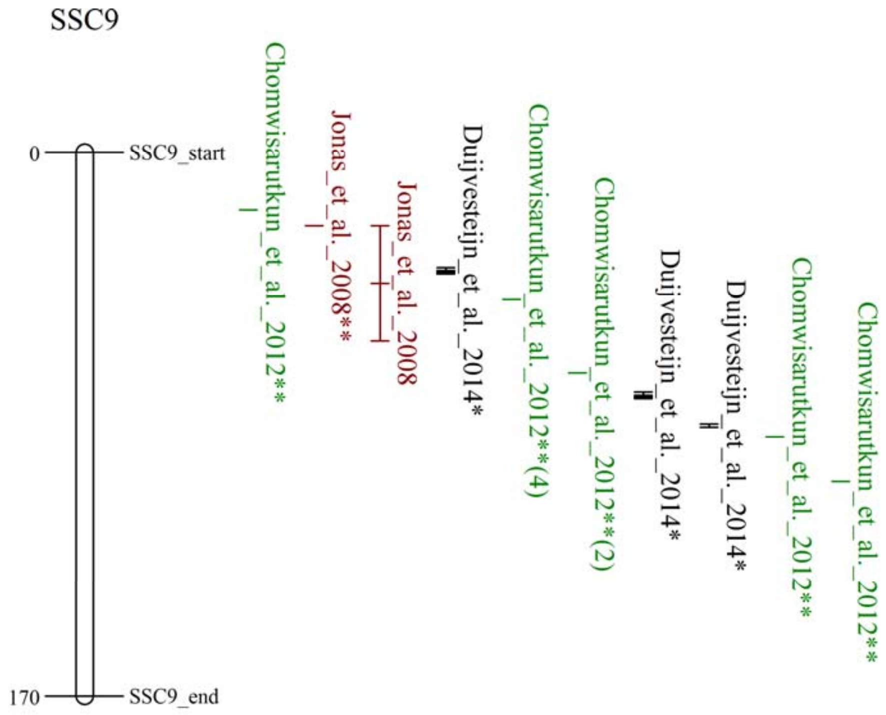
SSC9
0
SSC9_start
170
SSC9_end
Chomwisarutkun_et_al._2012**
Jonas_et_al._2008**
Jonas_et_al._2008
Duijvesteijn_et_al._2014*
Chomwisarutkun_et_al._2012**(4)
Chomwisarutkun_et_al._2012**(2)
Duijvesteijn_et_al._2014*
Duijvesteijn_et_al._2014*
Chomwisarutkun_et_al._2012**
Chomwisarutkun_et_al._2012**

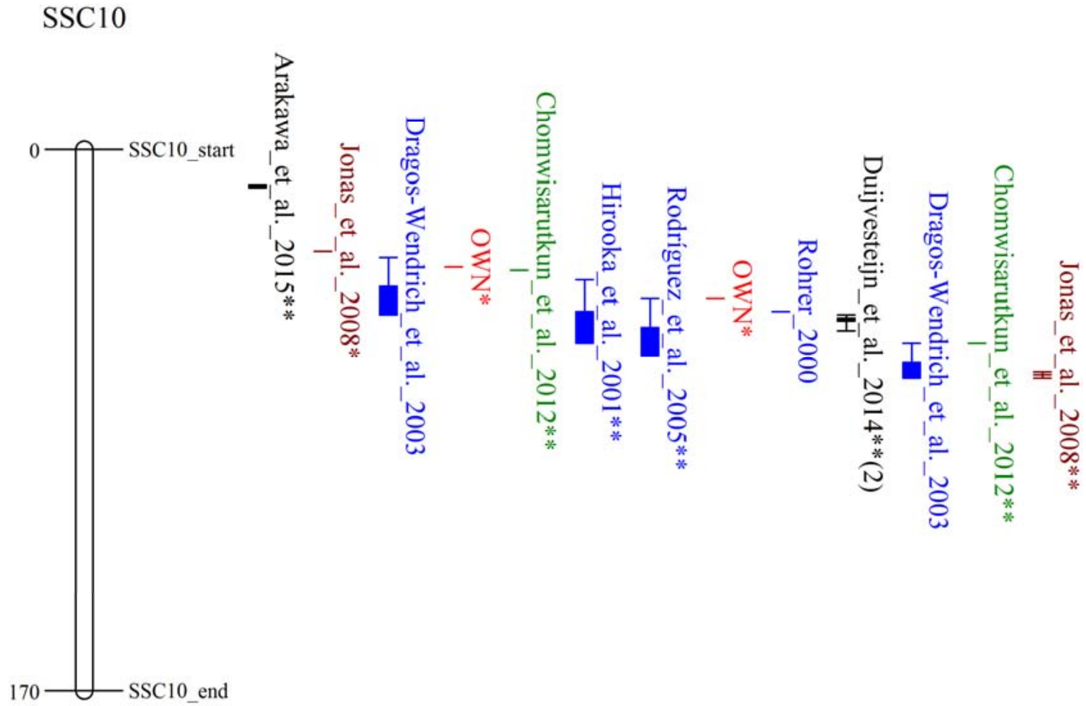
SSC10
0
SSC10_start
170
SSC10_end
Arakawa_et_al._2015**
Jonas_et_al._2008*
Dragos-Wendrich_et_al._2003
OWN*
Chomwisarutkun_et_al._2012**
Hirooka_et_al._2001**
Rodríguez_et_al._2005**
OWN*
Rohrer_2000
Duijvesteijn_et_al._2014**(2)
Dragos-Wendrich_et_al._2003
Chomwisarutkun_et_al._2012**
Jonas_et_al._2008**

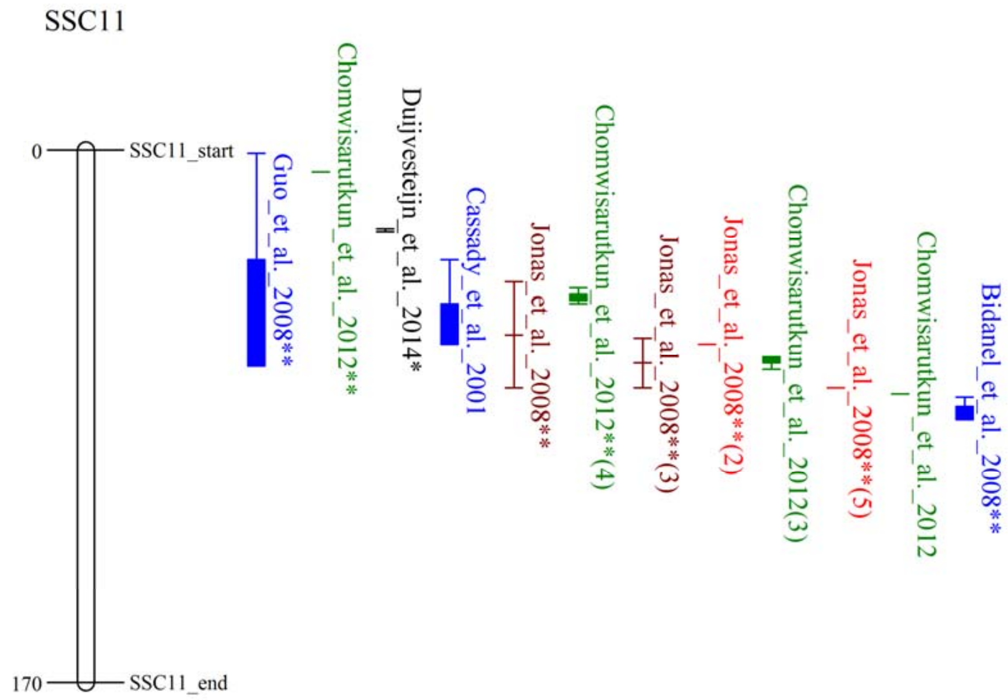

SSC11
0
SSC11_start
170
SSC11_end
Guo_et_al._2008**
Chomwisarutkun_et_al._2012**
Duijvesteijn_et_al._2014*
Cassady_et_al._2001
Jonas_et_al._2008**
Chomwisarutkun_et_al._2012**(4)
Jonas_et_al._2008**(3)
Jonas_et_al._2008**(2)
Chomwisarutkun_et_al._2012(3)
Jonas_et_al._2008**(5)
Chomwisarutkun_et_al._2012
Bidanel_et_al._2008**

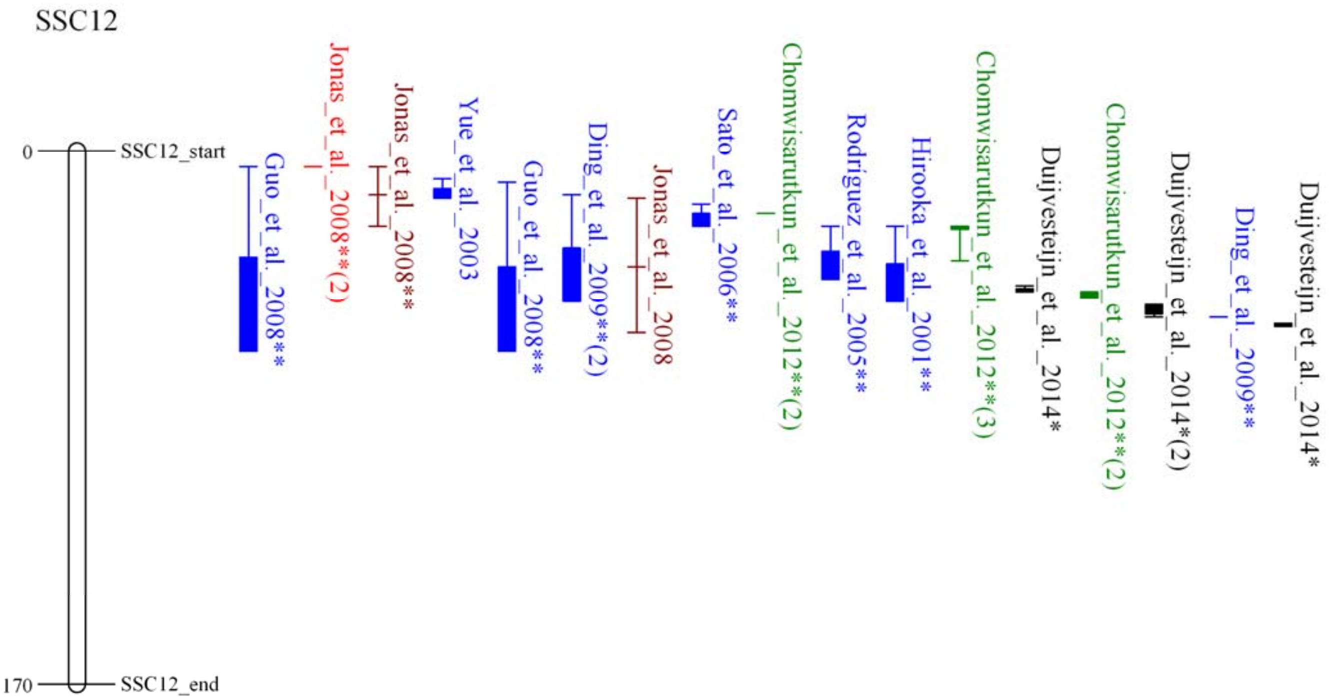
SSC12
0
SSC12_start
170
SSC12_end
Guo_et_al._2008**
Jonas_et_al._2008**(2)
Jonas_et_al._2008**
Yue_et_al._2003
Guo_et_al._2008**
Ding_et_al._2009**(2)
Jonas_et_al._2008
Sato_et_al._2006**
Chomwisarutkun_et_al._2012**(2)
Rodriguez_et_al._2005**
Hirooka_et_al._2001**
Chomwisarutkun_et_al._2012**(3)
Duijvesteijn_et_al._2014*
Chomwisarutkun_et_al._2012**(2)
Duijvesteijn_et_al._2014*(2)
Ding_et_al._2009**
Duijvesteijn_et_al._2014*

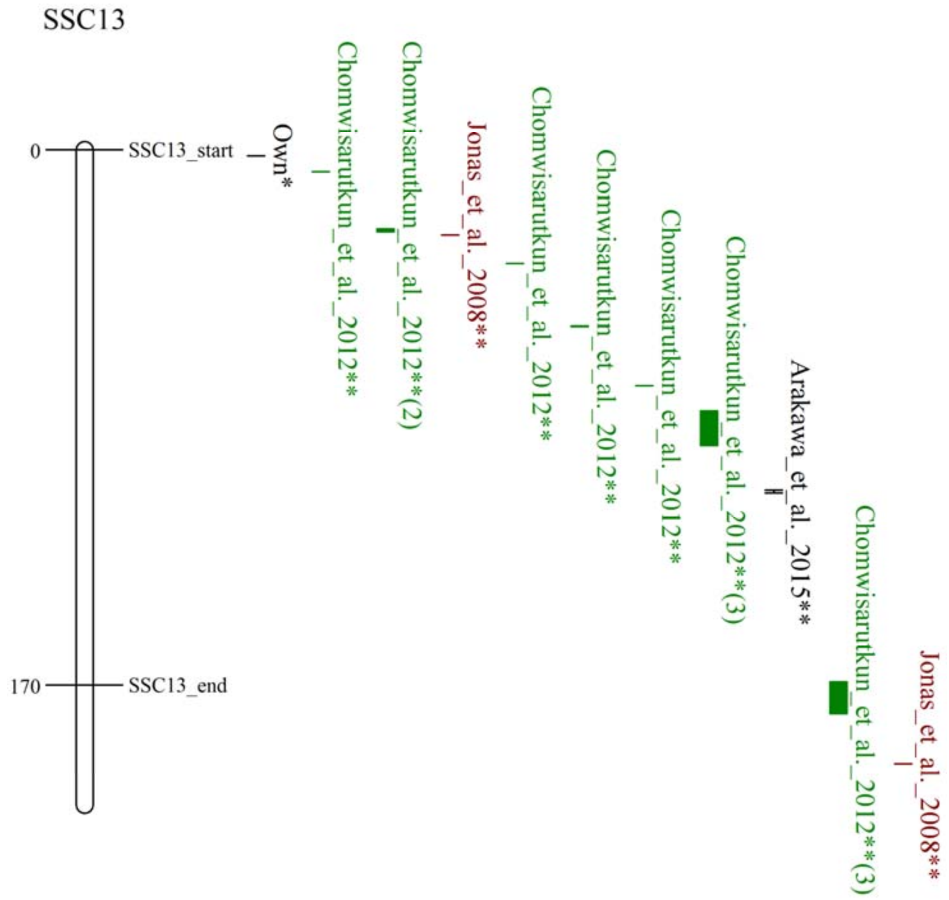

SSC13
0
SSC13_start
170
SSC13_end
Own*
Chomwisarutkun_et_al._2012**
Chomwisarutkun_et_al._2012**(2)
Jonas_et_al._2008**
Chomwisarutkun_et_al._2012**
Chomwisarutkun_et_al._2012**
Chomwisarutkun_et_al._2012**
Chomwisarutkun_et_al._2012**(3)
Arakawa_et_al._2015**
Chomwisarutkun_et_al._2012**(3)
Jonas_et_al._2008**

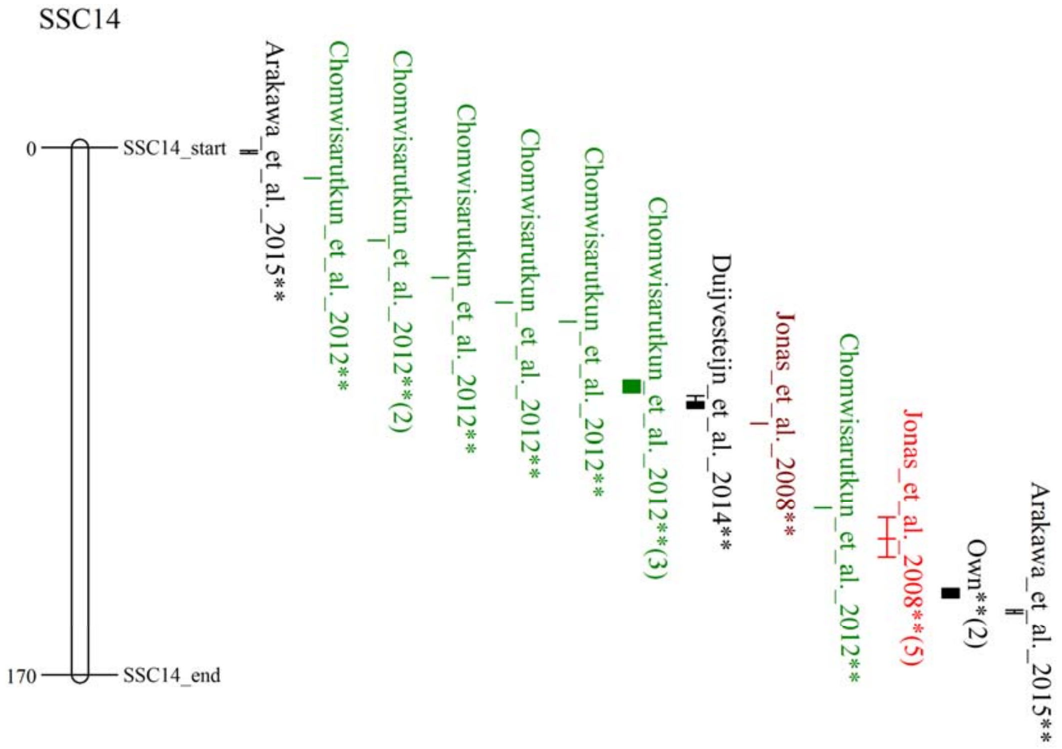
SSC14
0
SSC14_start
170
SSC14_end
Arakawa_et_al._2015**
Chomwisarutkun_et_al._2012**
Chomwisarutkun_et_al._2012**(2)
Chomwisarutkun_et_al._2012**
Chomwisarutkun_et_al._2012**
Chomwisarutkun_et_al._2012**
Chomwisarutkun_et_al._2012**(3)
Duijvesteijn_et_al._2014**
Jonas_et_al._2008**
Chomwisarutkun_et_al._2012**
Jonas_et_al._2008**(5)
Own**(2)
Arakawa_et_al._2015**

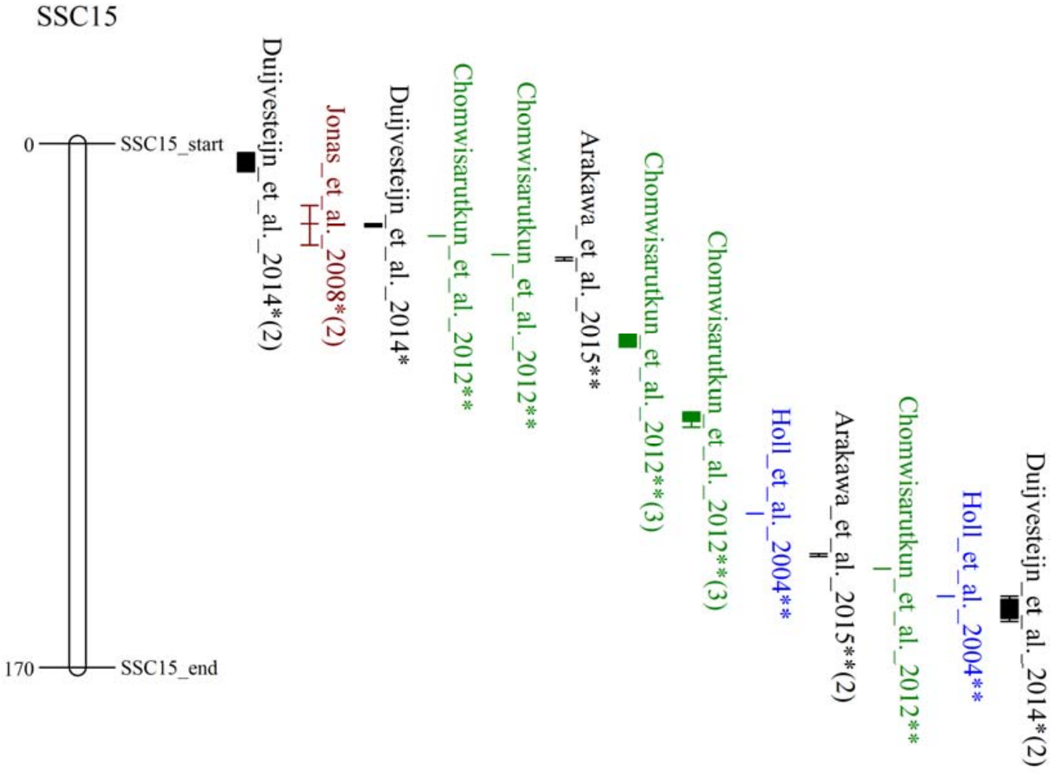

SSC15
0
SSC15_start
170
SSC15_end
Duijvesteijn_et_al._2014*(2)
Jonas_et_al._2008*(2)
Duijvesteijn_et_al._2014*
Chomwisarutkun_et_al._2012**
Chomwisarutkun_et_al._2012**
Arakawa_et_al._2015**
Chomwisarutkun_et_al._2012**(3)
Chomwisarutkun_et_al._2012**(3)
Holl_et_al._2004**
Arakawa_et_al._2015**(2)
Chomwisarutkun_et_al._2012**
Holl_et_al._2004**
Duijvesteijn_et_al._2014*(2)

SSC16

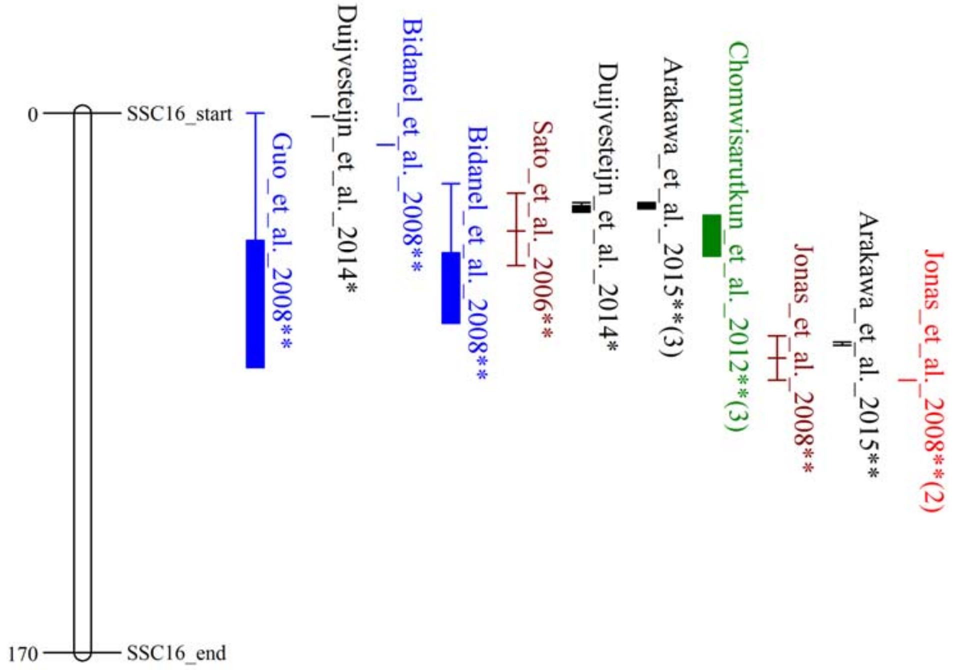
0
SSC16_start
170
SSC16_end
Guo_et_al._2008**
Duijvesteijn_et_al._2014*
Bidanel_et_al._2008**
Bidanel_et_al._2008**
Sato_et_al._2006**
Duijvesteijn_et_al._2014*
Arakawa_et_al._2015**(3)
Chomwisarutkun_et_al._2012**(3)
Jonas_et_al._2008**
Arakawa_et_al._2015**
Jonas_et_al._2008**(2)

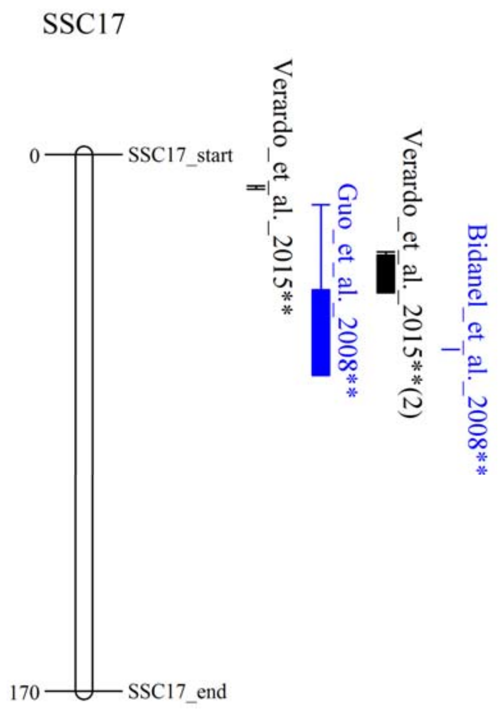
SSC17
0
SSC17_start
170
SSC17_end
Verardo_et_al._2015**
Guo_et_al._2008**
Verardo_et_al._2015**(2)
Bidanel_et_al._2008**

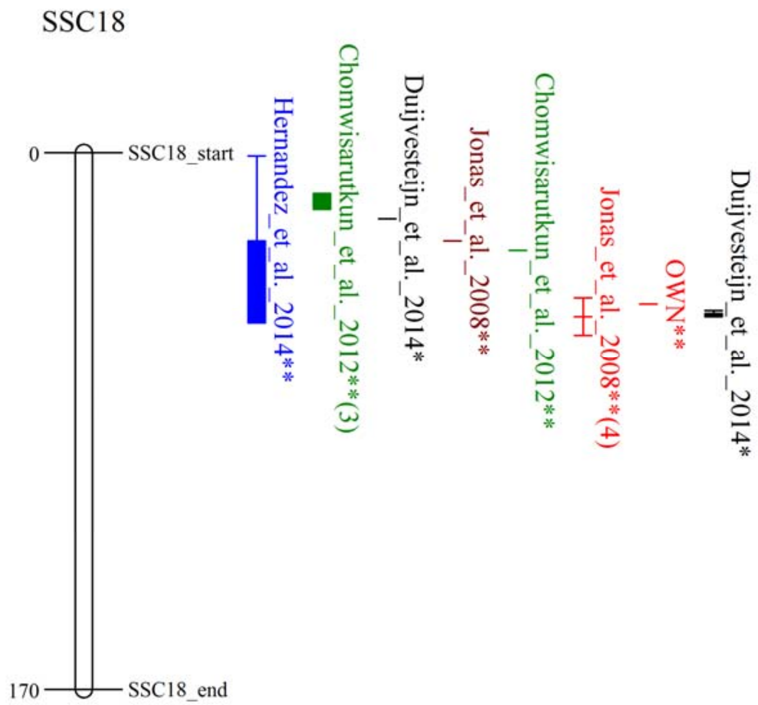
SSC18
0
SSC18_start
170
SSC18_end
Hernandez_et_al._2014**
Chomwisarutkun_et_al._2012**(3)
Duijvesteijn_et_al._2014*
Jonas_et_al._2008**
Chomwisarutkun_et_al._2012**
Jonas_et_al._2008**(4)
OWN**
Duijvesteijn_et_al._2014*

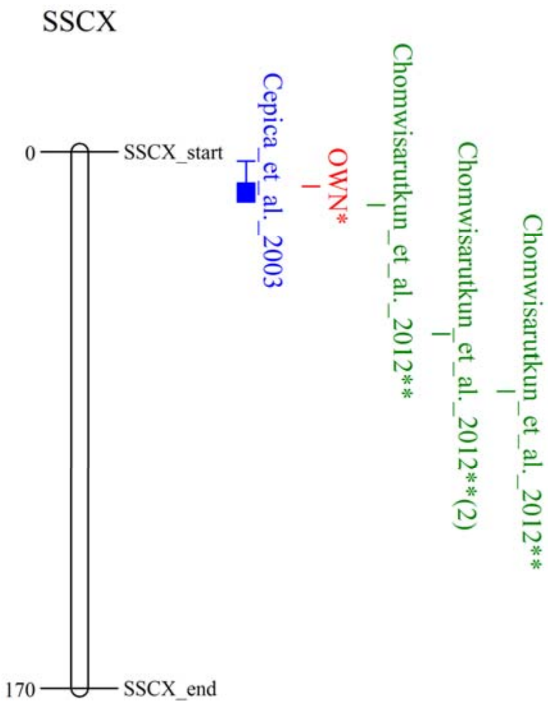
SSCX
0
SSCX_start
170
SSCX_end
Cepica_et_al._2003
OWN*
Chomwisarutkun_et_al._2012**
Chomwisarutkun_et_al._2012**(2)
Chomwisarutkun_et_al._2012**
